# Efficacy and safety of once-weekly basal insulin versus once-daily basal insulin in patients with type 2 diabetes: A systematic review and meta-analysis

**DOI:** 10.1097/MD.0000000000036308

**Published:** 2023-12-29

**Authors:** Xinxin Wang, Wei Xiao, Zhanpeng Liang, Shixiang Li, Qizhi Tang

**Affiliations:** a Affiliated Guangdong Hospital of Integrated Traditional Chinese and Western Medicine of Guangzhou University of Chinese Medicine, Nanhai District, Foshan City, Guangdong Province, China; b Department of Oncology, Zhongshan Hospital of Traditional Chinese Medicine Affiliated to Guangzhou University of Traditional Chinese Medicine, Guangdong Province, China; c School of Traditional Chinese Medicine, Jinan University, Tianhe District, Guangzhou City, Guangdong Province, China; d. Department of Endocrinology, Guangdong Provincial Hospital of Integrated Traditional Chinese and Western Medicine, Foshan City, Guangdong Province, the People’s Republic of China.

**Keywords:** meta-analysis, once-daily basal insulin, once-weekly basal insulin, systematic review, type 2 diabetes

## Abstract

**Background:**

Once-weekly insulin is expected to improve treatment compliance and durability and lead to better glycemic control. Several clinical trials on once-weekly insulin have recently been published. We conducted a systematic review and meta-analysis to investigate the efficacy and safety of once-weekly insulin versus once-daily insulin in type 2 diabetes (T2D).

**Methods:**

The following databases were searched for studies: PubMed, EMBASE, and Cochrane library (From January 1, 1946 to May 9, 2023). All randomized trials comparing weekly versus daily insulin in T2D were eligible for inclusion. Data analysis was performed using STATA 17.0 software (Stata Corporation, College Station, TX). The main outcomes and indexes included reduction in Hemoglobin A1c (HbA_1c_), fasting plasma glucose and bodyweight, proportion of patients achieving HbA_1c_ < 7%, time-in-range 70 to 180 mg/dL and adverse events.

**Results:**

This systematic review and meta-analysis included 7 randomized controlled studies involving 2391 patients (1347 receiving 1-week insulin and 1044 receiving 1-day insulin). Once-weekly insulin was not inferior to once-daily insulin in HbA_1c_ change [estimated treatment difference (ETD) = −0.05; 95% confidence intervals (CI): −0.14 to 0.04), HbA_1c_ **< **7% (odds ratio = 1.14; 95% CI: 0.87–1.50), fasting plasma glucose (ETD = 0.09; 95% CI: −0.19 to 0.36) and body weight loss (ETD = 0.27; 95% CI: −0.36 to 0.91). In terms of time-in-range 70 to 180 mg/dL, weekly insulin was superior to daily insulin (MTD = 3.84; 95% CI: 1.55–6.08). Icodec was associated with higher incidence of all adverse events (odds ratio = 1.20; 95% CI: 1.03–1.48; *P* = .024), but did not result in high risk of serious and severe adverse events. Moreover, icodec and Basal Insulin Fc did not result in higher incidence of hypoglycemia compared with insulin daily.

**Conclusion:**

Our meta-analysis found that insulin weekly was well tolerated and effective for glycemic control. Once-weekly insulin was not inferior to once-daily insulin in both efficacy and safety in T2D.

## 1. Introduction

The increasing number of people with diabetes worldwide not only poses a serious threat to human health, but also brings huge economic burden to society. As one of the 2 major drugs in the treatment of diabetes (insulin and oral hypoglycemic drugs), insulin has been a great boon for hundreds of millions of diabetic patients who need insulin treatment. Treatment of type 2 diabetes (T2D) often requires the addition of basal insulin as the disease progresses if the patient does not meet blood glucose targets.^[1]^ Compared with oral hypoglycemic drugs, insulin needs to be injected, thereby bringing inconvenience to many patients. According to the currently approved clinical application of insulin analogues, patients with diabetes often need to be injected at least once a day, possibly 2 to 4 times, and may also need to be combined with oral drugs for treatment, which mean complex treatment strategies and tedious self-blood glucose management.^[2]^ Clinical inertia is common in the management of T2D due to the need for frequent injections, and is often the reason why blood sugar is not effectively controlled.^[3–5]^ Although once-daily basal insulin analogues have solved some of these problems, long-term daily injection therapy is still a heavy burden on patients. Thus, reducing the number of injections may increase the acceptance and adherence of patients with T2D to insulin therapy, ultimately leading to improve blood sugar control. Weekly GLP1 injections improve treatment adherence and durability compared to daily injections, and these improvements may lead to better blood sugar control.^[6–9]^ With the successful marketing of “once a week” glucagon-like peptide-1 receptor agonist (GLP-1RA) weekly formulations, people are full of expectations for once-weekly basal insulin. Recent clinical studies have found a similar effect with once-weekly basal insulin.^[10,11]^ At present, the current research of basic insulin preparation mainly includes Basal Insulin Fc (BIF) and icodec 2 types. Randomized controlled studies of these 2 types of insulin show some promise to change the treatment landscape. To more fully evaluate the role of weekly basal insulin, we conducted a meta-analysis that pooled all current randomized controlled studies comparing the efficacy and safety of once-weekly basal insulin to once-daily basal insulin in patients with T2D.

## 2. Methods

This study was registered in the PROSPERO database (CRD42023425651) and was conducted according to the preferred reporting project for systematic review and meta-analysis (PRISMA) statement.^[12]^ The objective of this meta-analysis was to compare the efficacy and safety of once-weekly basal insulin with once-daily basal insulin in T2D.

### 2.1. Eligibility criteria

The study was independently screened by 2 authors. The inclusion criteria used to select studies in this meta-analysis were: Patients clinically diagnosed with T2D; Prospective Phase II or III randomized clinical trials comparing once-weekly basal insulin with once-daily basal insulin in T2D; Studies reporting at least one of the following results: reduction in hemoglobin A1c (HbA_1c_), fasting plasma glucose (FPG) and bodyweight, proportion of patients achieving HbA_1c_ < 7%, time-in-range 70 to 180 mg/dL (TIR) and adverse events (AEs). Exclusion criteria: Patients clinically diagnosed with type 1 diabetes mellitus; Non-randomized controlled studies, basic studies, retrospective studies, case reports, duplicate publications, and studies for which relevant data cannot be extracted.

### 2.2. Literature search

Randomized Control Trials comparing once-weekly insulin icodec versus once-daily insulin, in patients with T2D, were identified by computerized search of PubMed, Embase and Cochrane Library (From January 1, 1946 to May 9, 2023), using the following search terms: once-weekly insulin, once-daily insulin, Icodec, weekly basal Insulin Fc, BIF and type 2 diabetes. The detailed search strategy is as described in the Supplemental Digital Content, http://links.lww.com/MD/L249. There are no language or date restrictions on searches. If overlapping data exists, the most complete and updated report is selected for inclusion in this meta-analysis. Manually review references from all eligible studies to find other relevant studies.

### 2.3. Study selection and data extraction

Two experienced system reviewers independently screen records for eligibility. Any disagreements are resolved by consulting a third reviewer. Browse titles and abstracts to complete an initial selection, then browse the full text of potentially eligible articles, and select eligible articles based on preestablished criteria. Data were extracted using a prespecified data collection form, which included baseline characteristics, sample size and interventions used, changes in the number of assessable patients HbA_1c_ change, HbA_1c_ **< **7%, FPG, body weight loss, TIR, AEs, etc. Two system reviewers independently extract relevant data and resolve differences by consulting a third reviewer. When multiple articles contained overlapping patient series, we prioritized extraction of outcome data from the major articles with the largest sample size for early outcomes and the articles with the longest follow-up time for late outcomes.

### 2.4. Risk of bias

Two of our reviewers independently applied the Cochrane Collaboration tool to assess the quality of included trials in the following areas: random sequence generation, assignment hiding, blinding, incomplete results data, and selective results reporting.^[13]^ Differences were resolved by consulting a third reviewer.

### 2.5. Statistical analysis

Data were analyzed using STATA 17.0 software (Stata Corporation, College Station, TX). HbA_1c_ change, FPG, bodyweight and TIR were reported as estimated treatment difference (ETD) and corresponding 95% confidence intervals (CI). Proportion of patients achieving HbA_1c_ < 7% and AEs reported as odds ratio (OR) and 95% CI. Due to the heterogeneity among different study designs, methods and populations, in order to make the results more reliable, the random effects model was used in this study to combine relevant data. In addition, the Cochrane Q test and *I*^2^ test were used to evaluate heterogeneity among the studies. *I*^2^ higher than 50% is considered to be highly heterogeneous.^[14,15]^ Publication bias was assessed by funnel plot and Egger regression test.^[16]^ If heterogeneity was found, meta regression was performed to further explore the source of heterogeneity. Subgroup analyses of insulin type, duration of treatment, and presence or absence of basal insulin therapy were planned.

Statement: Our meta-analysis does not address the subject’s life, health, dignity, privacy, and other related issues. All analyses were based on previous published studies, thus no ethical approval or patient consent was required.

## 3. Result

### 3.1. Study identification and characteristics

**Figure 1.**
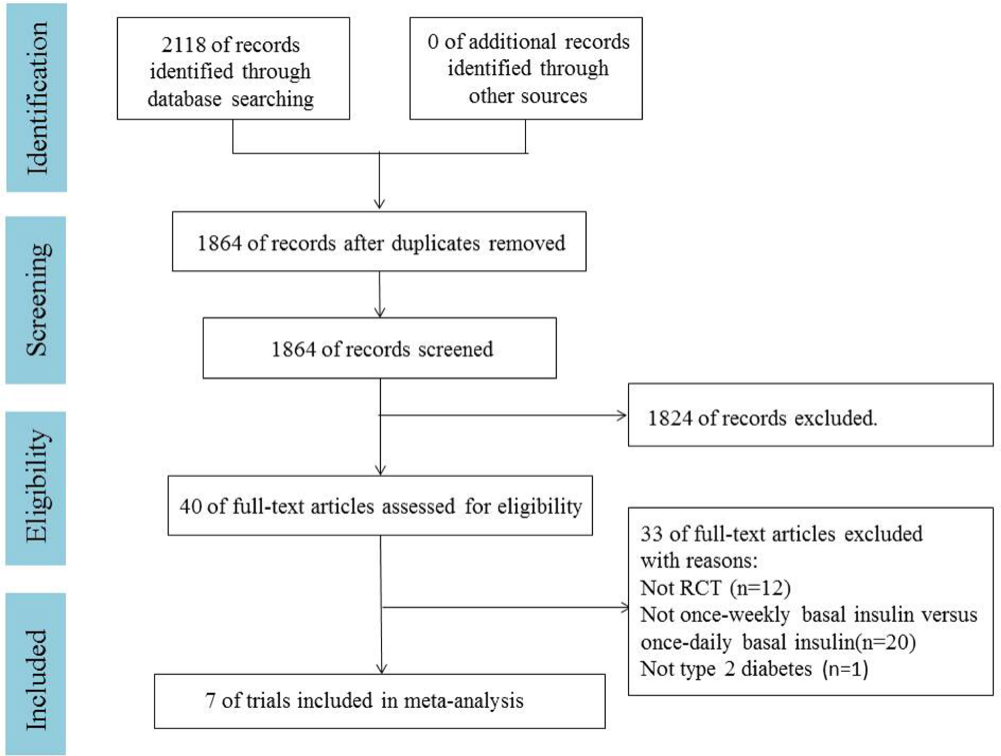
PRISMA flow diagram. RCT = randomized controlled trial.

A total of 2118 articles were retrieved from PubMed, EMBASE and Cochrane library. After removing 254 duplicates, a total of 1864 were eliminated by reading the title and abstract. Forty of them read the full text. Finally, this meta-analysis included 7 randomized controlled trials involving 2391 patients.^[10,11,17–21]^ A PRISMA flow chart describing study identification and selection is shown in Figure 1. Three trials included insulin-naive patients with T2D,^[17,19,20]^ while 4 trials included basal insulin-treated patients with T2D.^[10,11,18,21]^ Five trials evaluated the efficacy of Icodec^[10,17–19,21]^ and 2 trials evaluated BIF.^[11,20]^ One trial evaluated 3 different once-weekly insulin dosing schedules,^[19]^ and 2 trials evaluated 2 once-weekly insulin dosing schedules.^[11,18]^ Treatment lasted 16 weeks in 2 trials,^[18,19]^ 26 weeks in 4 trials,^[10,17,20,21]^ and 32 weeks in 1 trial^[11]^ (Table 1).

**Table 1:** Characteristics of included studies.

Author Year	Year Started	Study design	Patient characteristics	Treatment	Sample size	Preprandial Plasma glucose target	Duration	Primary Endpoint
Rosenstock2020	2018-11	Double blind	Insulin-naive Patients	Icodec (70U once per wk, adjusted ± 28 units/wk according to FBG) + metformin ± DPP4i	125	3.9–6.0 mmol/L	26 wk	HbA_1c_ change
				Glargine 10U once per d (adjusted ± 4 units/d according to FBG) + metformin ± DPP4i	122	3.9–6.0 mmol/L		
Bajaj2021	2019-05	Open label	Basal insulin-treated	Icodec (loading dose [first wk] + pretrial insulin daily doses × 7, adjusted ± 28 units/wk)	54	4.4–7.2 mmol/L	16 wk	TIR
				Icodec (pretrial insulin daily doses × 7, adjusted ± 28 units/wk according to FBG)	50	4.4–7.2 mmol/L		
				Glargine (the same dose as the pretrial insulin daily dose, adjusted ± 4 units/d according to FBG)	50	4.4–7.2 mmol/L		
Lingvay2021	2019-05	Open label	Insulin-naive Patients	Icodec (70U once per wk, adjusted ± 21 units/wk according to FBG)	51	4.4–7.2 mmol/L	16 wk	TIR
				Icodec (70U once per wk, adjusted ± 28 units/wk according to FBG)	51	4.4–7.2 mmol/L		
				Icodec (70U once per wk, adjusted ± 28 units/wk according to FBG)	52	3.9–6.0 mmol/L		
				Glargine (10U once per d, adjusted ± 4 units/d according to FBG)	51	4.4–7.2 mmol/L		
Bue-Valleskey2023	2020-07	Open label	Insulin-naive Patients	BIF (loading dose [first wk] + weekly dose which was based on baseline median FBG and body weight, dose titration was done to maintain FBG of < 100 mg/dL.)	143	4.4–5.6 mmol/L	26 wk	HbA_1c_ change
				Degludec (10U once per d, 2 unit increase for every 1 mmol/L increase)	135	4.4–5.6 mmol/L		
Tsimikas2023	2021-03	Open label	Basal insulin-treated	Icodec (pretrial insulin daily doses × 7 [first wk increased by 50%], adjusted 20 units/wk according to FBG)	263	4.4–7.2 mmol/L	26 wk	HbA_1c_ change
				Glargine (the same dose as the pretrial insulin daily dose, adjusted 3 units/d according to FBG)	263	4.4–7.2 mmol/L		
Frias2023	2018-11	Open label	Basal insulin-treated	BIF (loading dose followed by weekly dose of BIF based on the prior randomization basal insulin dose, dose titration was done to maintain FBG of < 140 mg/dL)	135	4.4–7.8 mmol/L	32 wk	HbA_1c_ change
				BIF (loading dose followed by weekly dose of BIF based on the prior randomization basal insulin dose, dose titration was done to maintain FBG of < 120 mg/dL adjusted according to FBG)	132	4.4–6.7 mmol/L		
				Degludec (the same dose as the total daily basal insulin dose, adjusted per modified Riddle algorithm)	132	4.4–5.6 mmol/L		
Mathieu2023	2021-05	Open label	Basal insulin-treated	Icodec (pretrial insulin daily doses × 7 [first wk increased by 50%], adjusted 20 units/wk according to FBG) ± non-insulin glucose-lowering agents + aspart 2-4 times daily	291	4.4–7.2 mmol/L	26 wk	HbA_1c_ change
				Glargine (the same dose as the pretrial insulin daily dose, adjusted 3 units/d according to FBG) ± non-insulin glucose-lowering agents + aspart 2–4 times daily	291	4.4–7.2 mmol/L		

BIF = basal insulin Fc, DPP4i = dipeptidyl peptidase 4 inhibitors, FPG = fasting plasma glucose, HbA_1c_, = Hemoglobin A1c.

### 3.2. Risk of bias assessment

Since all studies included were randomized, selection and loss bias were minimized. Most studies used central randomization for random allocation, and only 1 trials did not mention the method of allocation hiding.^[17]^ The main source of bias is that blindness was not used in most of the trials. A certain risk of bias may arise from the fact that blood glucose was not assessed blind in the study of Frias et al^[11]^ (Table S1, Supplemental Digital Content, http://links.lww.com/MD/K905).

### 3.3. Meta-analysis

#### 3.3.1. HbA1c change.

**Figure 2.**
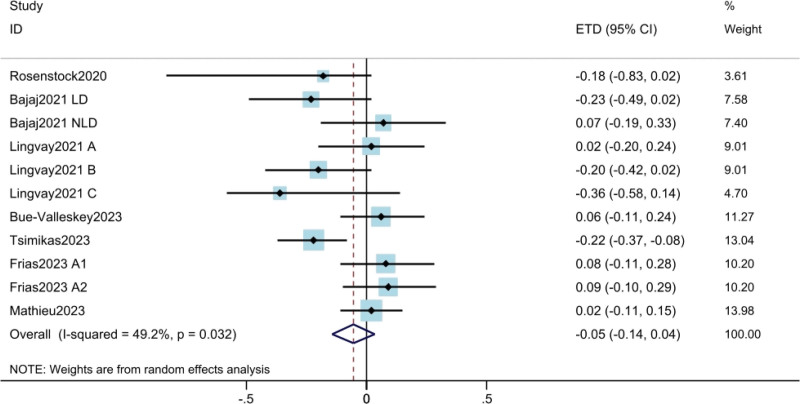
Assessment of HbA1c change. The diamond indicates best estimate of the true (pooled) outcome (with width indicating 95% CI). Due to the heterogeneity among different study designs, methods and populations, a random effects model was used. CI = confidence intervals, ETD = estimated treatment difference, HbA1c = hemoglobin A1c.

The results for HbA_1c_ came from 7 studies^[10,11,17–21]^ involving a total of 2391 patients. The results showed that weekly insulin dose was not inferior to daily insulin dose in HbA_1c_ change (ETD = −0.05; 95% CI: −0.14 to 0.04; *P* = .239) (Fig. 2). Moderate heterogeneity was found among the trials (*I*^2^ = 49.2%). The results of meta regression showed that insulin type, treatment duration, and basal insulin-treated were not the sources of heterogeneity (Table S2, Supplemental Digital Content, http://links.lww.com/MD/K906).

#### 3.3.2. Fasting plasma glucose.

**Figure 3.**
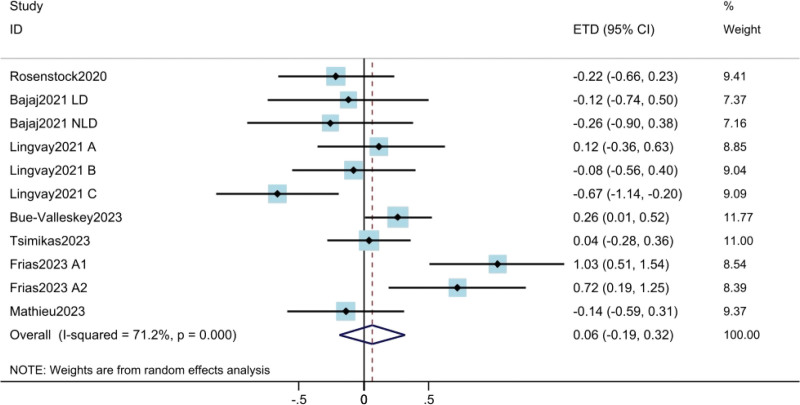
Assessment of fasting plasma glucose. The diamond indicates best estimate of the true (pooled) outcome (with width indicating 95% CI). Due to the heterogeneity among different study designs, methods and populations, a random effects model was used. CI = confidence intervals, ETD = estimated treatment difference.

The results of FPG were derived from 7 studies^[10,11,17–21]^ involving a total of 2391 patients. The results showed that weekly insulin was not inferior to daily insulin in FPG (ETD = 0.06; 95% CI: −0.19 to 0.32; *P* = .613) (Fig. 3). High heterogeneity was found among the trials (*I*^2^ = 71.2%). The results of meta regression suggested that the heterogeneity was due to insulin type and treatment duration.

#### 3.3..3. Body weight loss

**Figure 4.**
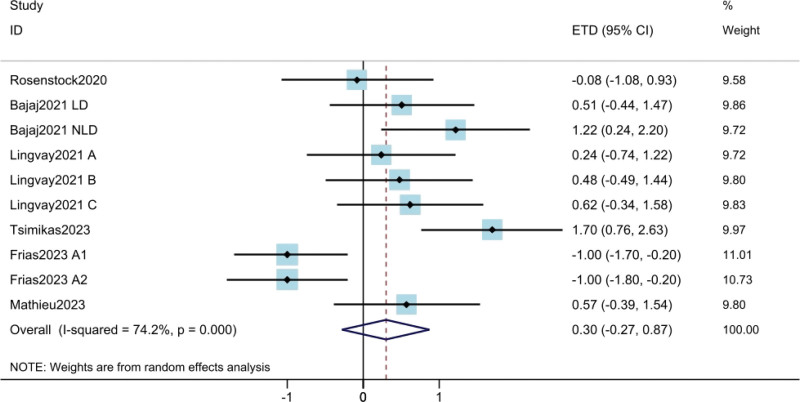
Assessment of body weight loss. The diamond indicates best estimate of the true (pooled) outcome (with width indicating 95% CI). Due to the heterogeneity among different study designs, methods and populations, a random effects model was used. CI = confidence intervals, ETD = estimated treatment difference.

The results of weight loss were derived from 6 studies^[10,11,17–19,21]^ involving a total of 2113 patients. Results showed no statistically significant difference in weight loss between weekly and daily insulin doses (ETD = 0.30; 95% CI: −0.27 to 0.87; *P* = .305) (Fig. 4). High heterogeneity was found among the trials (*I*^2^ = 74.2%). The results of meta regression suggested that the heterogeneity was due to insulin type and treatment duration (Table S2, Supplemental Digital Content, http://links.lww.com/MD/K906).

#### 3.3.4. HbA_1c_ < 7%.

**Figure 5.**
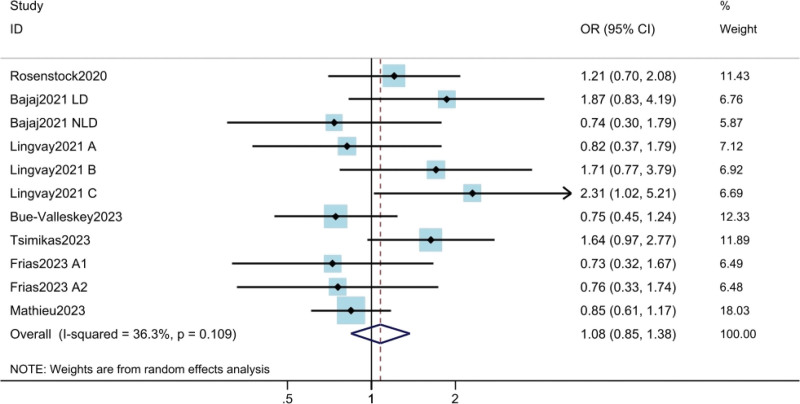
Assessment of HbA_1c_ < 7%. The diamond indicates best estimate of the true (pooled) outcome (with width indicating 95% CI). Due to the heterogeneity among different study designs, methods and populations, a random effects model was used. CI = confidence intervals, ETD = estimated treatment difference, HbA1c = hemoglobin A1c.

The results of HbA_1c_ <** **7% were obtained from 6 studies^[10,11,17–21]^ involving a total of 2391 patients. The results showed that there was no statistically significant difference in HbA_1c_ < 7% between weekly and daily insulin doses (OR = 1.08; 95% CI: 0.85–1.38; *P* = .530) (Fig. 5). Moderate heterogeneity was found among the trials (*I*^2^ = 36.3%). The results of meta regression suggested that the heterogeneity was due to insulin type.

#### 3.3.5. Time-in-range 70 to 180 mg/dL.

**Figure 6.**
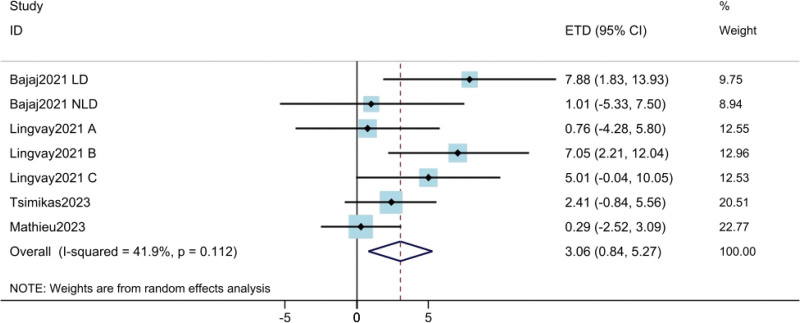
Assessment of time-in-range 70–180 mg/dL. The diamond indicates best estimate of the true (pooled) outcome (with width indicating 95% CI). Due to the heterogeneity among different study designs, methods and populations, a random effects model was used. CI = confidence intervals, ETD = estimated treatment difference.

The results of TIR were derived from 4 studies^[10,18,19,21]^ involving a total of 1467 patients. The results showed that compared with daily insulin dose, weekly insulin dose significantly improved TIR (MTD = 3.06; 95% CI: 0.84–5.27; *P* = .007) (Fig. 6). Moderate heterogeneity was found among the trials (*I*^2^ = 41.9%).

#### 3.3.6. Adverse events.

The results of adverse events came from 7 trials involving a total of 2389 patients.^[10,11,17–21]^ Due to the different types of adverse events reported for icodec and BIF, the data for icodec and BIF were combined separately (Table 2). Results showed that icodec was associated with higher incidence of all adverse events (OR = 1.20; 95% CI: 1.03–1.48; *P* = .024), but did not result in higher risk of serious and severe adverse events. In level 1 hypoglycemia, level 2 or 3 hypoglycemia, injection-site reaction, hypersensitivity event, and any adverse event probably or possibly related to basal insulin, there was no statistical difference between Icodec and once-daily insulin. On the other hand, there was no statistical difference between BIF and once-daily insulin in terms of level 1 hypoglycemia, nocturnal level 1 hypoglycemia, and level 2 hypoglycemia. However, BIF was associated with lower nocturnal level 2 hypoglycemia (OR = 0.68; 95% CI: 0.47–0.98; *P* = .036).

**Table 2:** Results of adverse events.

Outcome	Odd ratio (95% CI)	*I* ^2^	*P* value
Icodec			
Any adverse event	1.23 [1.03, 1.48]	0%	.024
Serious adverse event	1.01 [0.68, 1.51]	0%	.948
Severe adverse event	0.94 [0.55, 1.58]	0%	.803
Level 1 hypoglycemia	1.25 [0.84, 1.86]	67.8%	.280
Level 2 or 3 hypoglycemia	1.28 [0.73, 2.24]	56.1%	.393
Injection-site reaction	1.34 [0.66, 2.74]	0%	.414
Hypersensitivity event	1.32 [0.58, 2.99]	0	.503
Any adverse event probably or possibly related to basal insulin	1.32 [0.81, 2.15]	21.4%	.258
BIF			
Level 1 hypoglycemia	1.43 [0.98, 2.09]	0	.063
Nocturnal level 1 hypoglycemia	0.77 [0.50, 1.17]	30.2%	.225
Level 2 hypoglycemia	0.81 [0.56, 1.17]	12.3%	.260
Nocturnal level 2 hypoglycemia	0.68 [0.47, 0.98]	0	.036

BIF = Basal Insulin Fc, CI = confidence intervals.

### 3.4. Subgroup analysis

#### 3.4.1. Insulin type.

In HbA_1c_ Changes, the Icodec subgroup showed superior efficacy compared with once-daily insulin, but there was no statistical difference between the 2 groups in the BIF subgroup. There was no statistical difference in FPG among the icodec subgroups, but it was inferior to once-daily insulin in BIF. In addition, in HbA_1c_ < 7%, icodec and BIF were comparable to once-daily insulin. Interestingly, in terms of weight loss, the 2 subgroups had opposite benefits, with icodec being inferior to daily insulin and BIF being superior to daily insulin (Table 3 and Figure S1–S5, Supplemental Digital Content, http://links.lww.com/MD/K908, http://links.lww.com/MD/K909, http://links.lww.com/MD/K910, http://links.lww.com/MD/K911, http://links.lww.com/MD/K912).

**Table 3:** Results of subgroup analysis.

Subgroup		HbA_1c_ change (ETD,95% CI)	*P* value	FPG (MTD,95% CI)	*P* value	Body weight (ETD,95% CI)	*P* value	Time-in-range 70–180 mgdL (MTD,95% CI)	*P* value	HbA1c < 7% (OR,95% CI)	*P* value
Insulin type	Icodec	−0.11 [−0.22, −0.01]	.036	−0.14 [−0.31,0.03]	.102	0.67 [0.28, 1.06]	.001	NA	NA	1.23 [0.92, 1.65]	.160
	BIF	0.08 [−0.03, 0.18]	.172	0.63 [0.14, 1.12]	.012	−1.00 [−1.55, −0.45]	.000	NA	NA	0.74 [0.51, 1.09]	.132
Duration	16 wk	−0.06 [−0.21, 0.09]	.420	0.04 [−0.18, 0.26]	.704	0.74 [−0.28, 1.77]	.155	4.40 [1.55, 7.25]	.002	1.03 [0.74, 1.43]	.881
	26 wk	−0.12 [−0.27, 0.03]	.106	−0.21 [−0.49, 0.08]	.150	0.61 [0.18, 1.04]	.006	1.21 [−0.90,3.32]	.260	1.36 [0.87, 2.13]	.176
	32 wk	0.09 [−0.05, 0.22]	.227	0.88 [0.51, 1.25]	.000	−1.00 [−1.55, −0.45]	.000	NA	NA	0.74 [0.41, 1.34]	.320
Basal insulin-treated	Yes	−0.09 [−0.24, 0.06]	.242	−0.09 [0.43, −0.24]	.579	0.32 [−0.16, 0.81]	.193	2.26 [−0.48,5.00]	.105	1.17 [0.78, 1.75]	.446
	No	−0.03 [−0.15, 0.09]	.596	0.22 [−0.18, −0.62]	.289	0.31 [−0.63 1.24]	.521	4.30 [0.67,7.94]	.020	1.03 [0.73, 1.43]	.882

BIF = Basal Insulin Fc, CI = confidence intervals, ETD = estimated treatment difference, FPG = fasting plasma glucose, OR = odds ratio, HbA1c = Hemoglobin A1c, NA = not available, TIR = time-in-range 70–180 mg/dL.

#### 3.4.2. Treatment duration.

In HbA_1c_ changes and HbA_1c_ <** **7%, there was no statistical difference among the 3 subgroups. Moreover, there was no statistical difference in FPG for the 16-week and 26-week subgroups, but once-weekly insulin was inferior to once-daily insulin in the 32-week subgroup. Moreover, in terms of weight loss, there was no statistical significance in the 16-week treatment subgroup; the 26-week subgroup showed a superiority of weekly insulin, while the 32-week subgroup showed an inferiority of weekly insulin. On the contrary, there was no statistical significance in the 26-week treatment subgroup in TIR, but the 16-week subgroup showed a superiority of weekly insulin.

#### 3.4.3. Basal insulin-treated.

There were no significant differences in HbA_1c_ changes, HbA_1c_ <** **7%, FPG, weight loss and TIR in both of 2 subgroups.

### 3.5. Meta regression, sensitivity analysis, and publication bias

The results of meta regression suggested that the heterogeneity of most outcomes was due to insulin type (Table S2, Supplemental Digital Content, http://links.lww.com/MD/K906). Sensitivity analysis by removing 1 study at a time found that no 1 study affected the overall effect of the efficacy endpoint (Figure S6–10, Supplemental Digital Content, http://links.lww.com/MD/L239, http://links.lww.com/MD/L240, http://links.lww.com/MD/L241, http://links.lww.com/MD/L242, http://links.lww.com/MD/L243). Qualitative assessment was performed by assessing various measures for each individual study using the Cochrane Bias risk tool. Overall, these trials were considered to have a low-risk bias. The main source of bias was the lack of blinding in most of the included studies. The asymmetry of funnel plots was not significant except for weight loss (Figure S11–15, Supplemental Digital Content, http://links.lww.com/MD/L244, http://links.lww.com/MD/L245, http://links.lww.com/MD/L246, http://links.lww.com/MD/L247, http://links.lww.com/MD/L248). The Egger test results showed that there was publication bias in the outcome of body weight, and the potential of publication bias in all other efficacy endpoints was low (Table S3, Supplemental Digital Content, http://links.lww.com/MD/K907).

## 4. Discussion

Insulin is an indispensable treatment in T2D. Although oral hypoglycemic drugs provide great convenience, with the development of the disease, oral hypoglycemic drugs alone may no longer be able to effectively control blood glucose, and then additional insulin is needed to strengthen blood glucose control^[1]^ Diabetes is a chronic disease that requires long-term medication or insulin injections to control blood glucose and prevent complications. The inconvenience of frequent injections reduces patient compliance, as well as quality of life. Recently published clinical trials have shown promise in reducing the frequency of insulin injections to once a week. Therefore, we summarized all current randomized controlled trials and conducted a meta-analysis comparing the efficacy and safety of once-weekly insulin versus once-daily insulin. The results showed that once-weekly insulin was not inferior to insulin daily for T2D in HbA_1c_ changes, HbA_1c_ < 7%, FPG and weight loss. Notably, this non-inferior glycemic effect was not associated with increased risk of hypoglycemia. In terms of TIR, weekly insulin was superior to daily insulin. Our results demonstrated that once-weekly insulin was similar to once-daily insulin in terms of hypoglycemic risk, suggesting that once-weekly insulin was not inferior to once-daily insulin in terms of efficacy or safety. A previous meta-analysis of 3 trials found that once-weekly Insulin Icodec was associated with a small reduction in HbA_1c_, as well as higher Time with Glucose in Range. However, this meta-analysis included only 453 patients.^[22]^ Our meta-analysis included the recently updated Phase 2 and III clinical trials involving a total of 2391 patients, and conducted a subgroup analysis to explore the impact of prior insulin-treated, insulin type, and treatment duration on the outcome. Therefore, our results are more reliable and instructive.

Icodec is a basal insulin analogue whose strong, reversible binding to albumin and reduced insulin receptor affinity to slow clearance, leading to the formation of a circulating albumin binding library for insulin icodec. Therefore, it has a half-life of about 1 week and is suitable for once a-week administration.^[23–25]^ ONWARDS 2 and ONWARDS 4 are the first 2 Phase III trial to publish clinical data showing that icode is safe and efficacious in patients with T2D suboptimally controlled with basal insulin.^[10,21]^ In these studies, icodec showed non-inferior glycemic control compared with insulin glargine as measured by HbA_1c_ change from baseline to study end point. A previous, double-blind, randomized controlled trial of insulin-naive T2D patients showed similar results. This trial was more rigorous with the fasting blood glucose target.^[17]^ In addition, Novo Nordisk conducted ONWARDS1, 3, and 5 to evaluate the efficacy and safety of icodec in insulin-naive patients with T2D.^[26]^ However, as the full data of these 3 trials have not been published, they were not included in our meta-analysis. The results of these 3 studies indicate that icodec was not inferior to insulin daily in reducing HbA_1c_, which was consistent with the results of our meta-analysis. BIF, the second once-weekly insulin in development, is a novel fusion protein that binds a single chain insulin variant to the Fc domain of human immunoglobulin G.^[27,28]^ Unlike icodec, BIF has a longer half-life of 17 days. In 2 Phase II trials, BIF was administered using mg steps instead of international units of insulin to help establish clinically correct mg to international units conversion factors in all relevant populations. Phase II clinical trials have shown that BIF is safe and effective in insulin-naive and insulin-treated patients.^[11,20]^ Because of the long half-life, icodec and BIF also take longer to reach steady state. Thus, in insulin-treated patients, both icodec and BIF were given a loading dose, based on the results of the Phase 1 study, to reach homeostasis concentrations more quickly and minimize transient hyperglycemia. Bajaj et al^[18]^ evaluated the efficacy and safety of loaded dose and unloaded dose icodec versus once-daily insulin in insulin-treated patients. The results showed that TIR changes were greater in the loaded dose group than in the unloaded dose group (15.4% vs 8.6%) and there was no increased risk of hypoglycemia.

Some limitations should be taken into account when interpreting our results. In HbA_1c_, FPG, body weight loss, TIR and HbA_1c_ < 7% of the analyses, there was some degree of statistical heterogeneity, as measured by *I*^2^ statistics. Therefore, we conducted meta regression to explore the source of heterogeneity. The results showed that the heterogeneity was mainly due to insulin type. In order to better analyze heterogeneity, subgroup analysis was performed. Subgroup analysis of insulin type found icodec was superior to BIF in HbA1c change, FPG, and HbA_1c_ < 7%, but was inferior to BIF in weight loss. Upon further review of the included studies, it was found that the reason for this difference may be that the fasting glucose target of degludec in Frias et al^[11]^ study was ≤ 5.6 mmol/L, which was more stringent than that of BIF, while the fasting glucose target of icodec was consistent with that of once-daily insulin. In any insulin therapy, due to its physiological role as an anabolic hormone that promotes the uptake and storage of glucose by cells, there is always an inherent delicate balance between achieving good blood sugar control, hypoglycemia, and weight change. So this difference could partly explain why icodec outperformed BIF in blood glucose control and the opposite in weight loss. In the subgroup analysis of treatment duration, the 32 week subgroup included only Frias et al,^[11]^ so we found a similar situation where BIF was inferior to insulin daily in terms of FPG. In addition, to assess the impact of prior insulin therapy, a subgroup analysis was performed to explore the difference between weekly doses of insulin in initial and treated patients. The results showed that there were no significant differences in HbA_1c_, HbA_1c_** **<** **7%, FPG, body weight loss and TIR between the 2 subgroups.

In terms of adverse events, icodec was associated with higher risk of all adverse events, but not more serious adverse events. In addition, icodec did not lead to a more severe incidence of hypoglycemia, nor did level 2 or 3 hypoglycemia. No level 3 hypoglycemic events due to icodec were observed in other studies except for 1 level 3 hypoglycemic event in the study by Rosenstock et al^[17]^ and 4 in the study by Mathieu et al^[21]^. On the other hand, BIF did not result in more severe hypoglycemic events. Interestingly, BIF reduced the incidence of nocturnal level 2 hypoglycemia. As mentioned earlier, Frias et al^[11]^ fasting glucose targets for degludec were more stringent than those for BIF. Our meta-analysis found that icodec and BIF were safe and feasible compared to once-daily insulin.

By reducing the number of basal insulin injections from at least 365 to 52 per year, once-weekly insulin significantly reduces the burden of daily insulin injections, increases treatment compliance and durability, and further improves glycemic control and long-term clinical outcomes in type 2 diabetes, as demonstrated by weekly GLP-1RA.^[6–9]^ In addition, basal insulin and GLP-1RA have a synergistic effect that can further improve blood glucose control and reduce the adverse side effects of each ingredient, such as weight gain, hypoglycemia and gastrointestinal symptoms.^[29–31]^ Therefore, the combination of weekly insulin and weekly GLP-1RA is a promising strategy. Future studies could explore the feasibility of this combination.

There are some limitations to our study. First of all, most experiments are open label designs. Secondly, some results were highly heterogeneous, but we conducted meta regression and subgroup analysis to fully explore the sources of heterogeneity and used a random effects model to reduce bias. Third, insulin schedules and fasting glucose targets were not identical across trials, which could lead to some bias.

## 5. Conclusion

The results of this meta-analysis of 7 randomized controlled trials (2391 participants) showed that once-weekly insulin was not inferior to once-daily insulin for T2D in HbA_1c_ change, HbA_1c_ <** **7%, FPG and weight loss. In terms of TIR, once-weekly insulin was superior to once-daily insulin. Moreover, the incidence of hypoglycemia was similar and the safety was comparable. Therefore, once-weekly insulin is safe and feasible in T2D.

## Author contributions

**Conceptualization:** Zhanpeng Liang.

**Data curation:** Xinxin Wang, Wei Xiao.

**Formal analysis:** Xinxin Wang, Wei Xiao.

**Funding acquisition:** Zhanpeng Liang, Shixiang Li, Qizhi Tang.

**Investigation:** Xinxin Wang, Wei Xiao, Zhanpeng Liang, Qizhi Tang.

**Methodology:** Xinxin Wang, Wei Xiao, Zhanpeng Liang.

**Project administration:** Wei Xiao.

**Resources:** Xinxin Wang.

**Software:** Xinxin Wang, Wei Xiao, Zhanpeng Liang.

**Supervision:** Shixiang Li, Qizhi Tang.

**Validation:** Shixiang Li, Qizhi Tang.

**Visualization:** Xinxin Wang, Wei Xiao, Shixiang Li, Qizhi Tang.

**Writing – original draft:** Xinxin Wang, Wei Xiao.

**Writing – review & editing:** Zhanpeng Liang, Qizhi Tang.

## Supplementary Material






































