# Supplementary material for: Efficacy and safety of once-weekly basal insulin versus once-daily basal insulin in patients with type 2 diabetes: A systematic review and meta-analysis
Source: Medicine (Baltimore). 2023 Dec 29;102(52):e36308. doi: 10.1097/MD.0000000000036308 (PMC10754560; doi:10.1097/MD.0000000000036308)
Supplement: Supplementary file 1 [file medi-102-e36308-s001.docx]

**eMethods.** Search Strategy

**PubMed < updated to 2023-05-09> Search Strategy (302)**

#1 "Diabetes Mellitus, Type 2"[Mesh])

#2 Diabetes Mellitus, Noninsulin-Dependent[Title/Abstract] OR Diabetes Mellitus, Ketosis-Resistant[Title/Abstract] OR Diabetes Mellitus, Ketosis Resistant[Title/Abstract] OR Ketosis-Resistant Diabetes Mellitus[Title/Abstract] OR Diabetes Mellitus, Non Insulin Dependent[Title/Abstract] OR Diabetes Mellitus, Non Insulin Dependent[Title/Abstract] OR Diabetes Mellitus, Non Insulin Dependent[Title/Abstract] OR Diabetes Mellitus, Stable[Title/Abstract] OR Stable Diabetes Mellitus[Title/Abstract] OR Diabetes Mellitus, Type II[Title/Abstract] OR NIDDM[Title/Abstract] OR Diabetes Mellitus, Noninsulin Dependent[Title/Abstract] OR Diabetes Mellitus, Noninsulin Dependent[Title/Abstract] OR Diabetes Mellitus, Maturity Onset[Title/Abstract] OR Diabetes Mellitus, Maturity Onset[Title/Abstract] OR Diabetes Mellitus, Maturity Onset[Title/Abstract] OR MODY[Title/Abstract] OR Diabetes Mellitus, Slow-Onset[Title/Abstract] OR Diabetes Mellitus, Slow-Onset[Title/Abstract] OR Diabetes Mellitus, Slow-Onset[Title/Abstract] OR Diabetes Mellitus, Slow-Onset[Title/Abstract] OR Maturity-Onset Diabetes[Title/Abstract] OR Diabetes, Maturity-Onset[Title/Abstract] OR Diabetes, Maturity-Onset[Title/Abstract] OR Diabetes, Maturity-Onset[Title/Abstract] OR Diabetes, Type 2[Title/Abstract] OR Diabetes Mellitus, Adult-Onset[Title/Abstract] OR Diabetes Mellitus, Adult-Onset[Title/Abstract] OR Diabetes Mellitus, Adult Onset[Title/Abstract]

#3 #1 OR #2

#4 Weekly OR once a week OR once per week OR once each week

#5 "Insulin"[Mesh] OR (Insulin, Regular[Title/Abstract] OR Insulin, Regular[Title/Abstract] OR Soluble Insulin[Title/Abstract] OR Insulin, Soluble[Title/Abstract] OR Basal Insulin Insulin A Chain[Title/Abstract] OR Insulin A Chain[Title/Abstract] OR Insulin, Sodium[Title/Abstract] OR Insulin, Sodium[Title/Abstract] OR Iletin[Title/Abstract] OR Insulin B Chain[Title/Abstract] OR Insulin B Chain OR[Title/Abstract]

#6 #4 AND #5

#7 Basal Insulin Fc OR BIF OR icodec

#8 #6 OR #7

#9 Daily OR [once a day](javascript:;) OR once per day OR once each day

#10 #5 AND #9

#11 Glargine OR Degludec

#12 #10 OR #11

#13 #3 AND #8 AND #12

**Embase < updated to 2023-05-09> Search Strategy（1072）**

#1 'non insulin dependent diabetes mellitus'/exp

#2 'type 2 diabetes':ab,ti OR 'diabetes mellitus, noninsulin-dependent':ab,ti OR 'diabetes mellitus, ketosis-resistant':ab,ti OR 'diabetes mellitus, ketosis resistant':ab,ti OR 'ketosis-resistant diabetes mellitus':ab,ti OR 'diabetes mellitus, non insulin dependent':ab,ti OR 'diabetes mellitus, stable':ab,ti OR 'stable diabetes mellitus':ab,ti OR 'diabetes mellitus, type ii':ab,ti OR niddm:ab,ti OR 'diabetes mellitus, noninsulin dependent':ab,ti OR 'diabetes mellitus, maturity onset':ab,ti OR mody:ab,ti OR 'diabetes mellitus, slow-onset':ab,ti OR 'maturity-onset diabetes':ab,ti OR 'diabetes, maturity-onset':ab,ti OR 'diabetes, type 2':ab,ti OR 'diabetes mellitus, adult-onset':ab,ti OR 'diabetes mellitus, adult onset':ab,ti

#3 #1 OR #2

#4 weekly OR 'once a week' OR (once AND a AND week) OR 'once per week' OR (once AND per AND week) OR 'once each week' OR (once AND each AND week)

#5 'insulin'/exp

#6 'insulin, regular':ab,ti OR 'soluble insulin':ab,ti OR 'insulin, soluble':ab,ti OR 'insulin a chain':ab,ti OR 'insulin, sodium':ab,ti OR iletin:ab,ti OR 'insulin b chain':ab,ti

#7 #5 AND #6

#8 'basal insulin fc':ab,ti OR bif:ab,ti OR icodec:ab,ti

#9 #7 OR #8

#10 daily OR 'once a day' OR (once AND a AND ('day'/exp OR day)) OR 'once per day' OR (once AND per AND ('day'/exp OR day)) OR 'once each day' OR (once AND each AND ('day'/exp OR day))

#11 #7 AND #10

#12 Glargine:ab,ti OR Degludec:ab,ti

#13 #11 OR #12

#14 #3 AND #9 AND #13

**Cochrane library < updated to 2023-05-09>（744）**

#1 MeSH descriptor: [Diabetes Mellitus, Type 2] explode all trees

#2 (Diabetes Mellitus, Noninsulin-Dependent OR Diabetes Mellitus, Ketosis-Resistant OR Diabetes Mellitus, Ketosis Resistant OR Ketosis-Resistant Diabetes Mellitus OR Diabetes Mellitus, Non Insulin Dependent OR Diabetes Mellitus, Non Insulin Dependent OR Diabetes Mellitus, Non Insulin Dependent OR Diabetes Mellitus, Stable OR Stable Diabetes Mellitus OR Diabetes Mellitus, Type II OR NIDDM OR Diabetes Mellitus, Noninsulin Dependent OR Diabetes Mellitus, Noninsulin Dependent OR Diabetes Mellitus, Maturity Onset OR Diabetes Mellitus, Maturity Onset OR Diabetes Mellitus, Maturity Onset OR MODY OR Diabetes Mellitus, Slow-Onset OR Diabetes Mellitus, Slow-Onset OR Diabetes Mellitus, Slow-Onset OR Diabetes Mellitus, Slow-Onset OR Maturity-Onset Diabetes OR Diabetes, Maturity-Onset OR Diabetes, Maturity-Onset OR Diabetes, Maturity-Onset OR Diabetes, Type 2 OR Diabetes Mellitus, Adult-Onset OR Diabetes Mellitus, Adult-Onset OR Diabetes Mellitus, Adult Onset):ti,ab,kw

#3 #1 OR #2

#4 (Weekly OR once a week OR once per week OR once each week):ti,ab,kw

#5 MeSH descriptor: [Insulins] in all MeSH products

#6 (Insulin, Regular OR Insulin, Regular OR Soluble Insulin OR Insulin, Soluble OR Insulin A Chain OR Insulin A Chain OR Insulin, Sodium OR Insulin, Sodium OR Iletin OR Insulin B Chain OR Insulin B Chain):ti,ab,kw

#7 #5 OR #6

#8 #4 AND #7

#9 (Basal Insulin Fc OR BIF OR icodec):ti,ab,kw

#10 #8 OR #9

#11 (Daily OR once a day OR once per day OR once each day):ti,ab,kw

#12 #7 AND #11

#13 (Glargine OR Degludec):ti,ab,kw

#14 #13 OR #12

#15 #3 AND #10 AND #14
