# Supplementary material for: Efficacy and safety of once-weekly basal insulin versus once-daily basal insulin in patients with type 2 diabetes: A systematic review and meta-analysis
Source: Medicine (Baltimore). 2023 Dec 29;102(52):e36308. doi: 10.1097/MD.0000000000036308 (PMC10754560; doi:10.1097/MD.0000000000036308)
Supplement: Supplementary file 2 [file medi-102-e36308-s002.docx]

**eTable 1.** Cochrane Risk of Bias Assessment Among Individual Studies

| **Domain** | Rosenstock2020 | Baja2021 | Lingvay2021 | Bue-Valleskey2023 | Tsimikas2023 | Frias2023 | Mathieu2023 |
| --- | --- | --- | --- | --- | --- | --- | --- |
| **Random Sequence**  **Generation** | Low risk | Low risk  (IWRS) | Low risk  (IWRS) | Low risk  (IWRS) | Low risk  (IWRS) | Low risk  (IWRS) | Low risk  (IWRS) |
| **Allocation Concealment** | Unclear | Low risk  (IWRS) | Low risk  (IWRS) | Low risk  (IWRS) | Low risk  (IWRS) | Low risk  (IWRS) | Low risk  (IWRS) |
| **Blinding of Participants** | Low risk | High risk | High risk | High risk | High risk | High risk | High risk |
| **Blinding of Personnel** | Low risk | High risk | High risk | High risk | High risk | High risk | High risk |
| **Blinding of Outcome Assessment** | Low risk | Low risk | Low risk | Low risk | Low risk | High risk | Unclear |
| **Incomplete Outcome**  **Data** | Low risk | Low risk | Low risk | Low risk | Low risk | Low risk | Low risk |
| **Selective Outcome**  **Reporting** | Low risk | Low risk | Low risk | Low risk | Low risk | Low risk | Low risk |
| **Other Sources**  **of Bias** | Low risk | Low risk | Low risk | Low risk | Low risk | Low risk | Low risk |

IWRS, interactive web-response system.
