# Supplementary material for: Efficacy and safety of once-weekly basal insulin versus once-daily basal insulin in patients with type 2 diabetes: A systematic review and meta-analysis
Source: Medicine (Baltimore). 2023 Dec 29;102(52):e36308. doi: 10.1097/MD.0000000000036308 (PMC10754560; doi:10.1097/MD.0000000000036308)
Supplement: Supplementary file 3 [file medi-102-e36308-s003.docx]

**eTable2.** Results of Meta-regression

|  | | HbA_1c_ change | FPG | Body weight | TIR | HbA_1c_ < 7% |
| --- | --- | --- | --- | --- | --- | --- |
| **Insulin type** | | 0.054 | 0.006 | 0.004 | NA | 0.002 |
| **Duration** | 32week | reference | reference | reference | NA | reference |
|  | 26week | 0.257 | 0.009 | 0.006 | reference | 0.478 |
|  | 16week | 0.132 | 0.004 | 0.006 | 0.165 | 0.155 |
| **Basal insulin treated** | | 0.235 | 0.133 | 0.914 | 0.429 | 0.996 |
