# Supplementary figures and images for: Efficacy and safety of once-weekly basal insulin versus once-daily basal insulin in patients with type 2 diabetes: A systematic review and meta-analysis
Source: Medicine (Baltimore). 2023 Dec 29;102(52):e36308. doi: 10.1097/MD.0000000000036308 (PMC10754560; doi:10.1097/MD.0000000000036308)

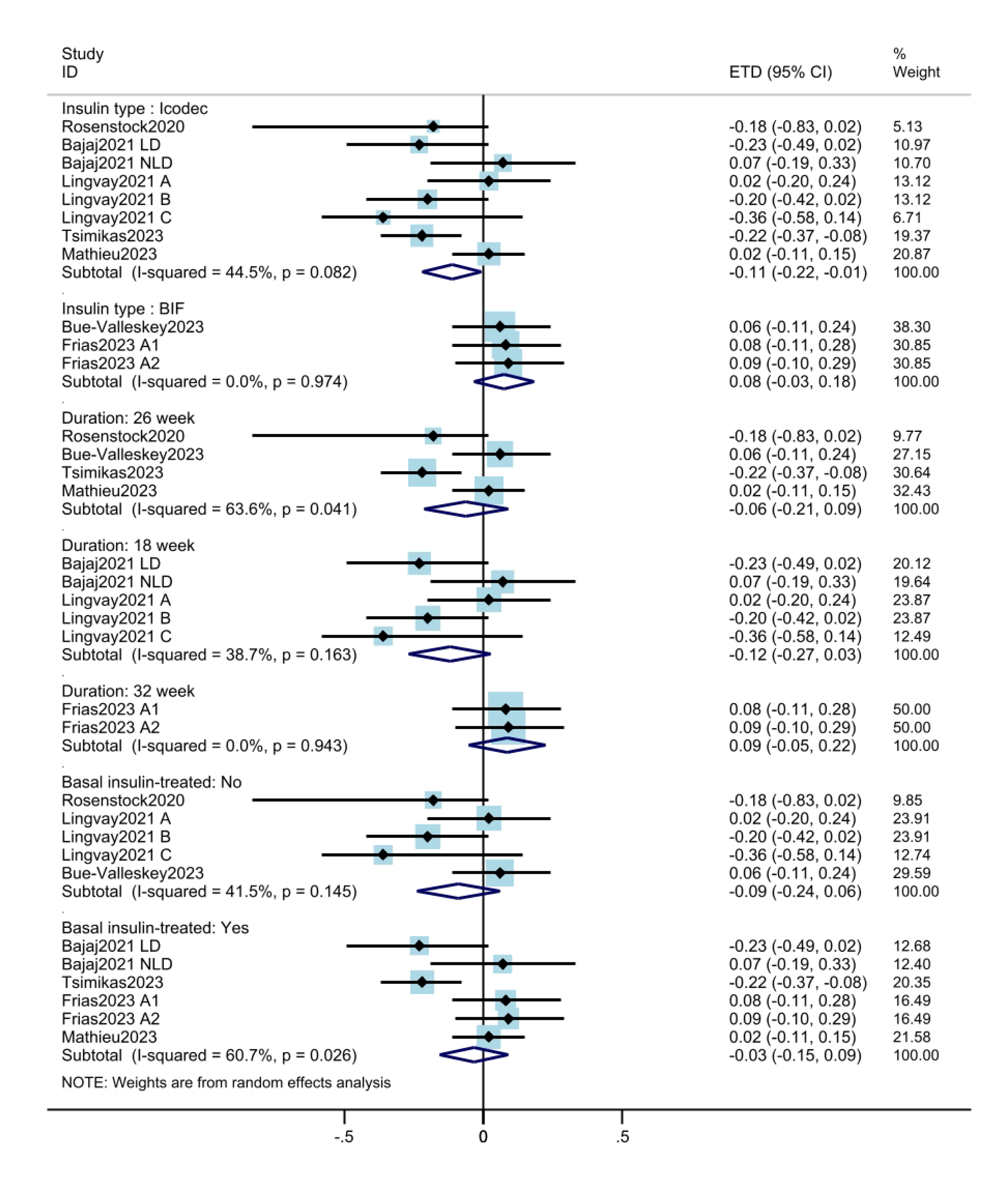

Supplement: Supplementary file 4 [file medi-102-e36308-s004.tif]

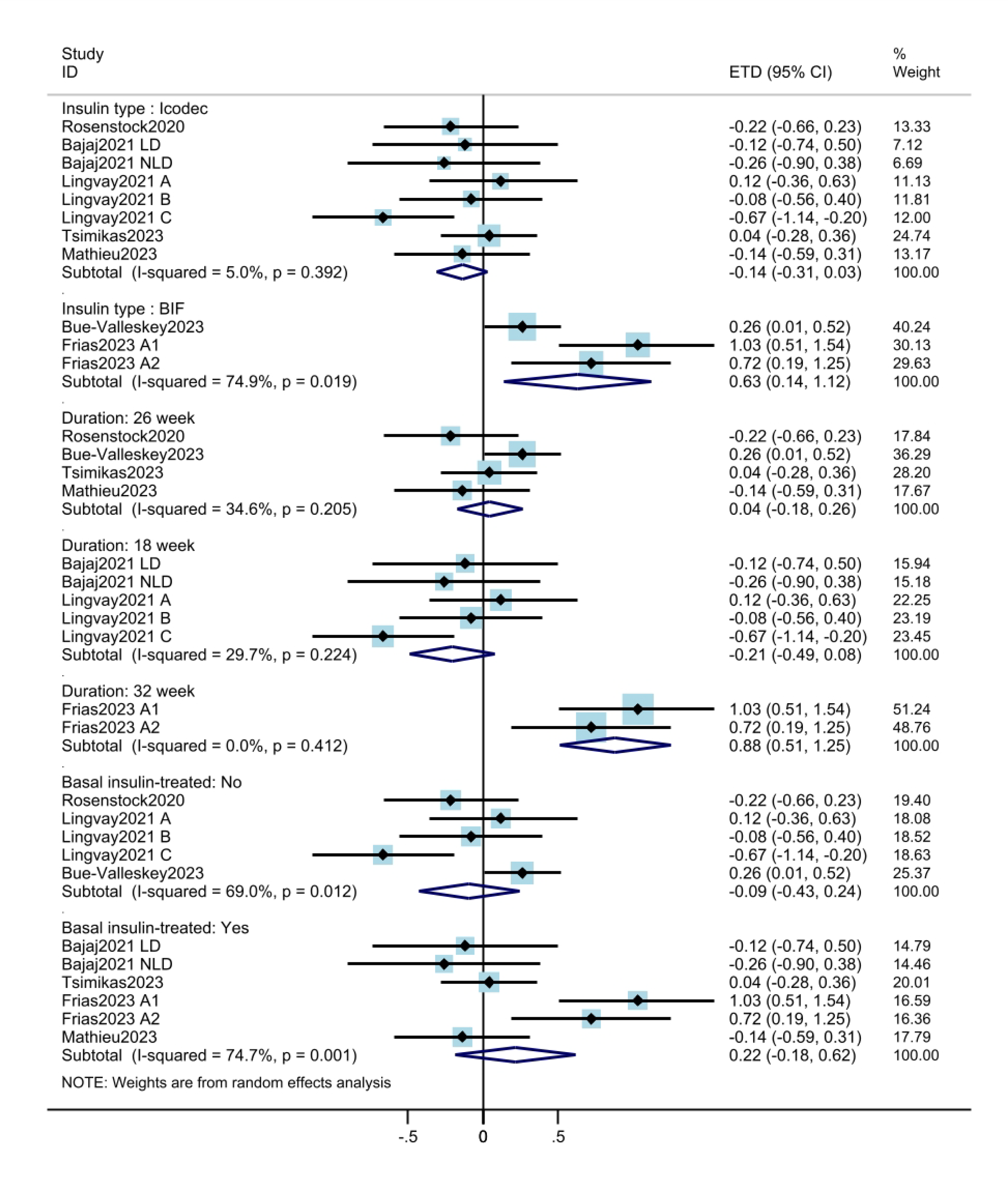

Supplement: Supplementary file 5 [file medi-102-e36308-s005.tif]

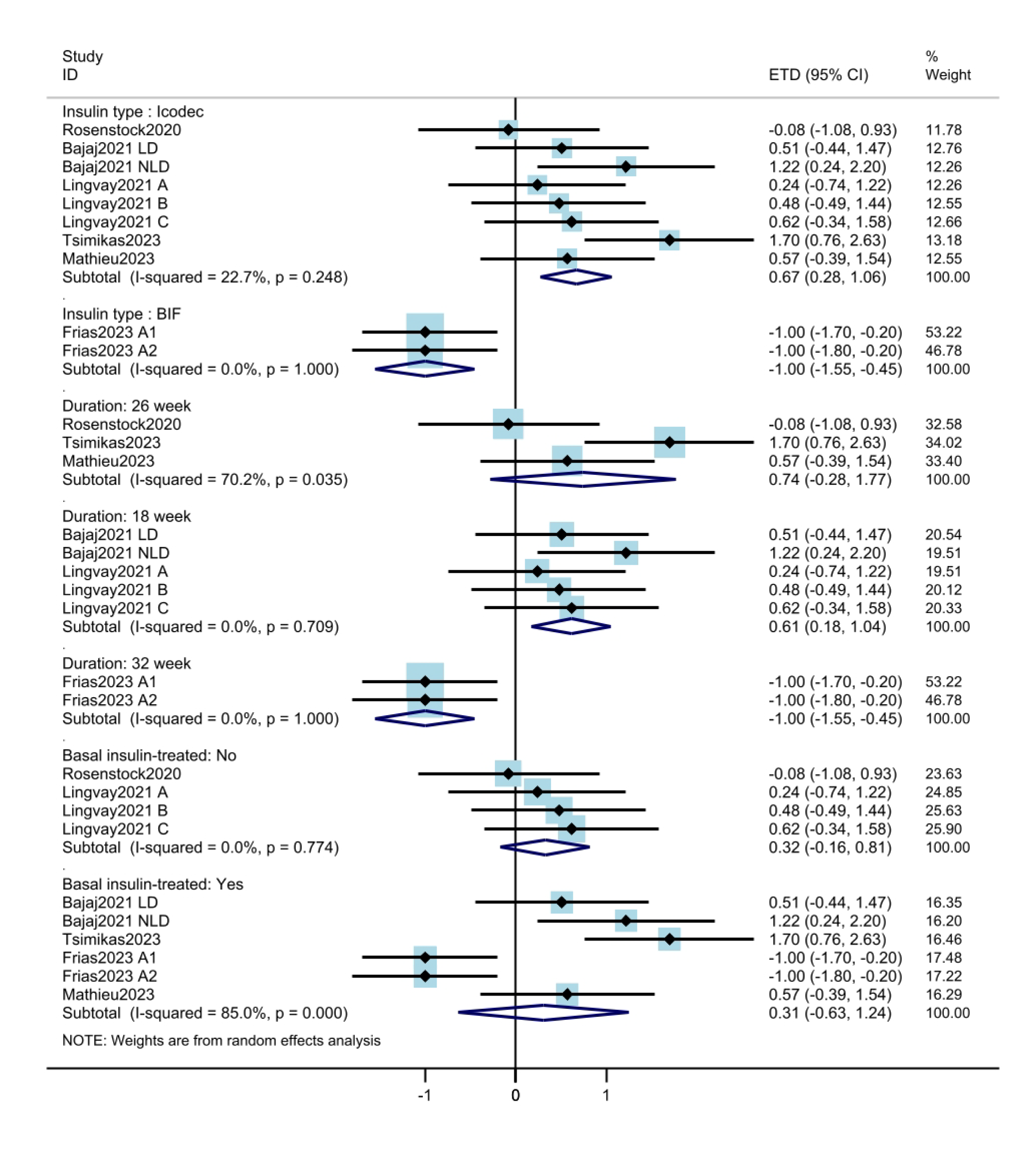

Supplement: Supplementary file 6 [file medi-102-e36308-s006.tif]

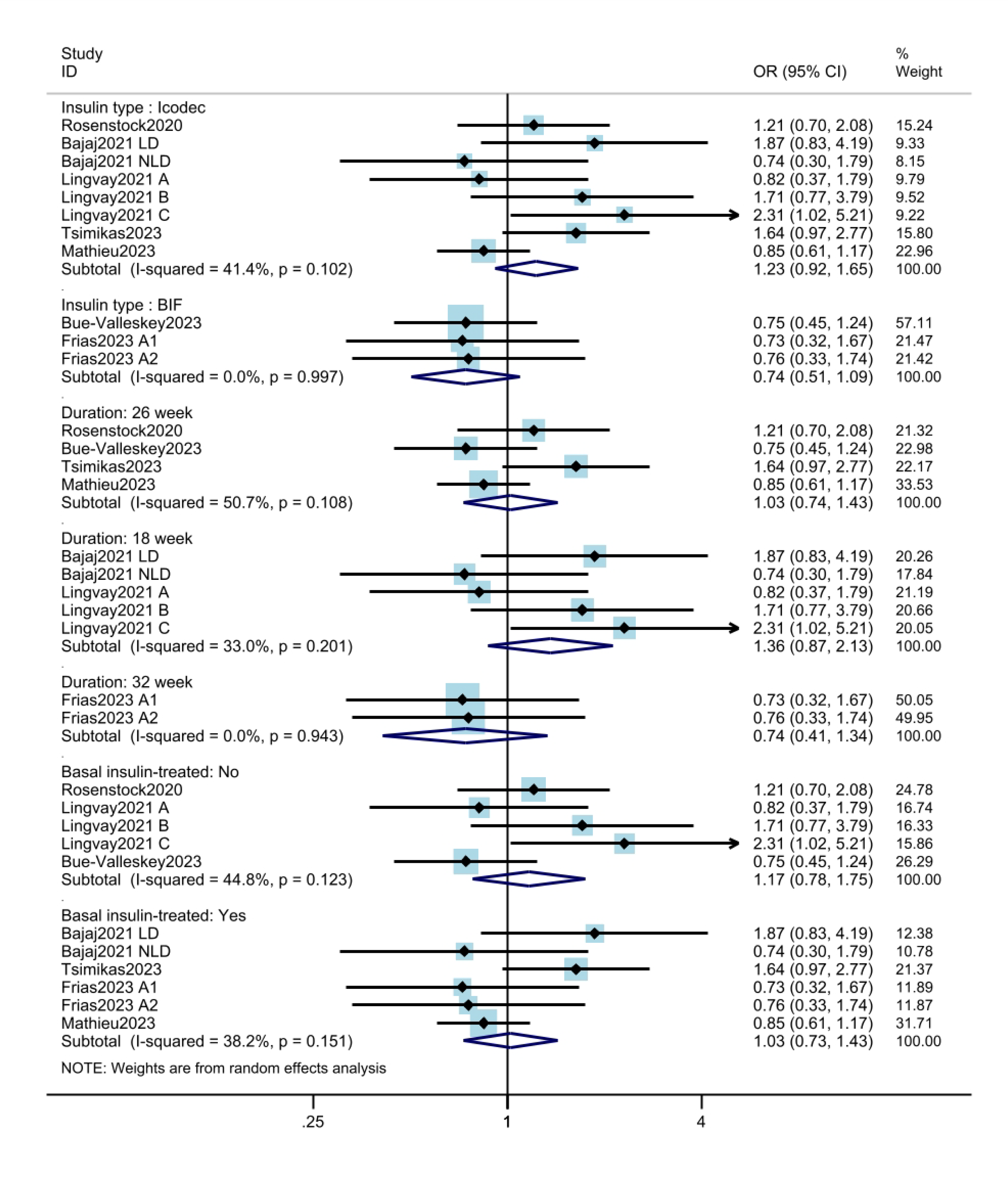

Supplement: Supplementary file 7 [file medi-102-e36308-s007.tif]

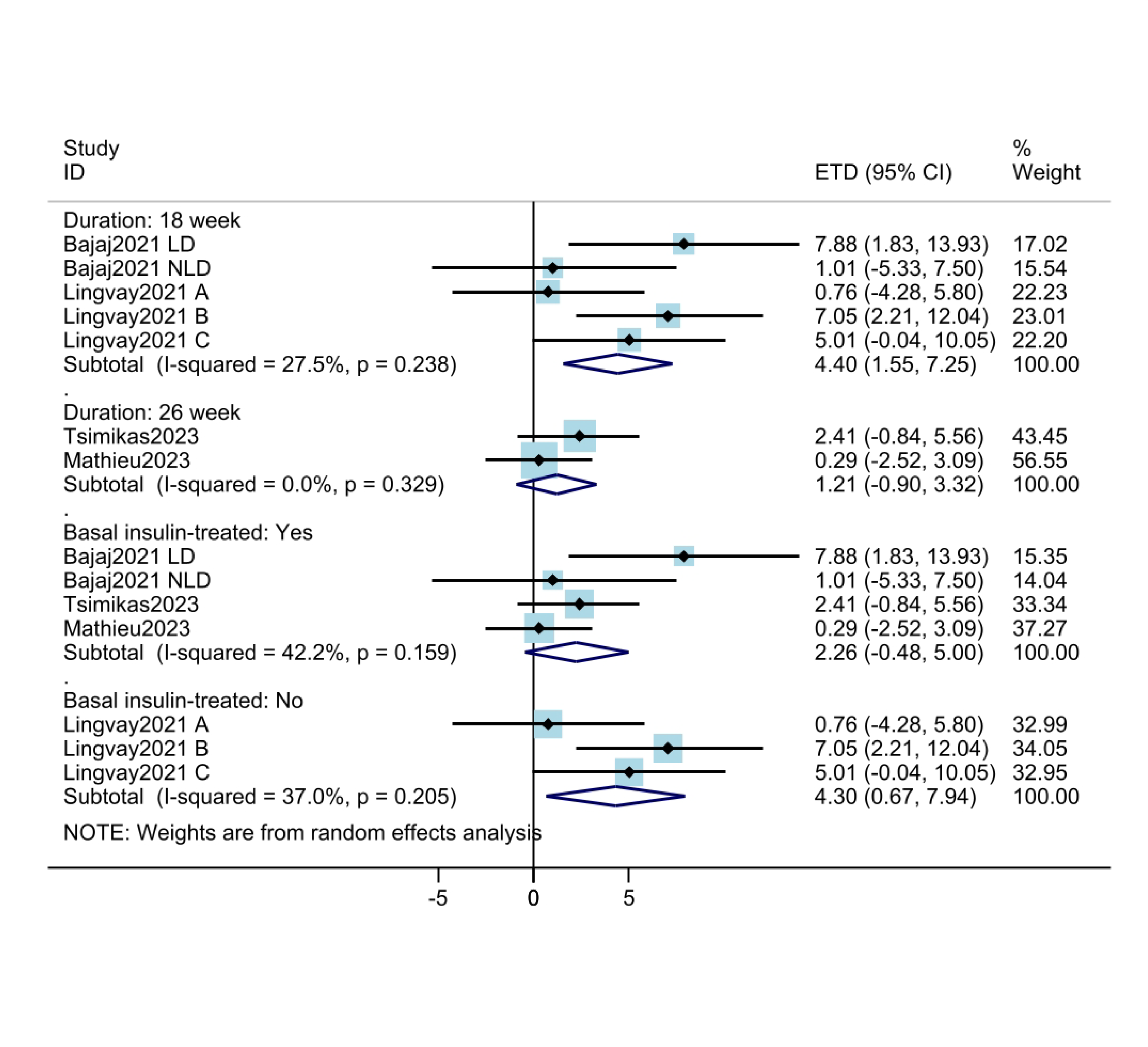

Supplement: Supplementary file 8 [file medi-102-e36308-s008.tif]

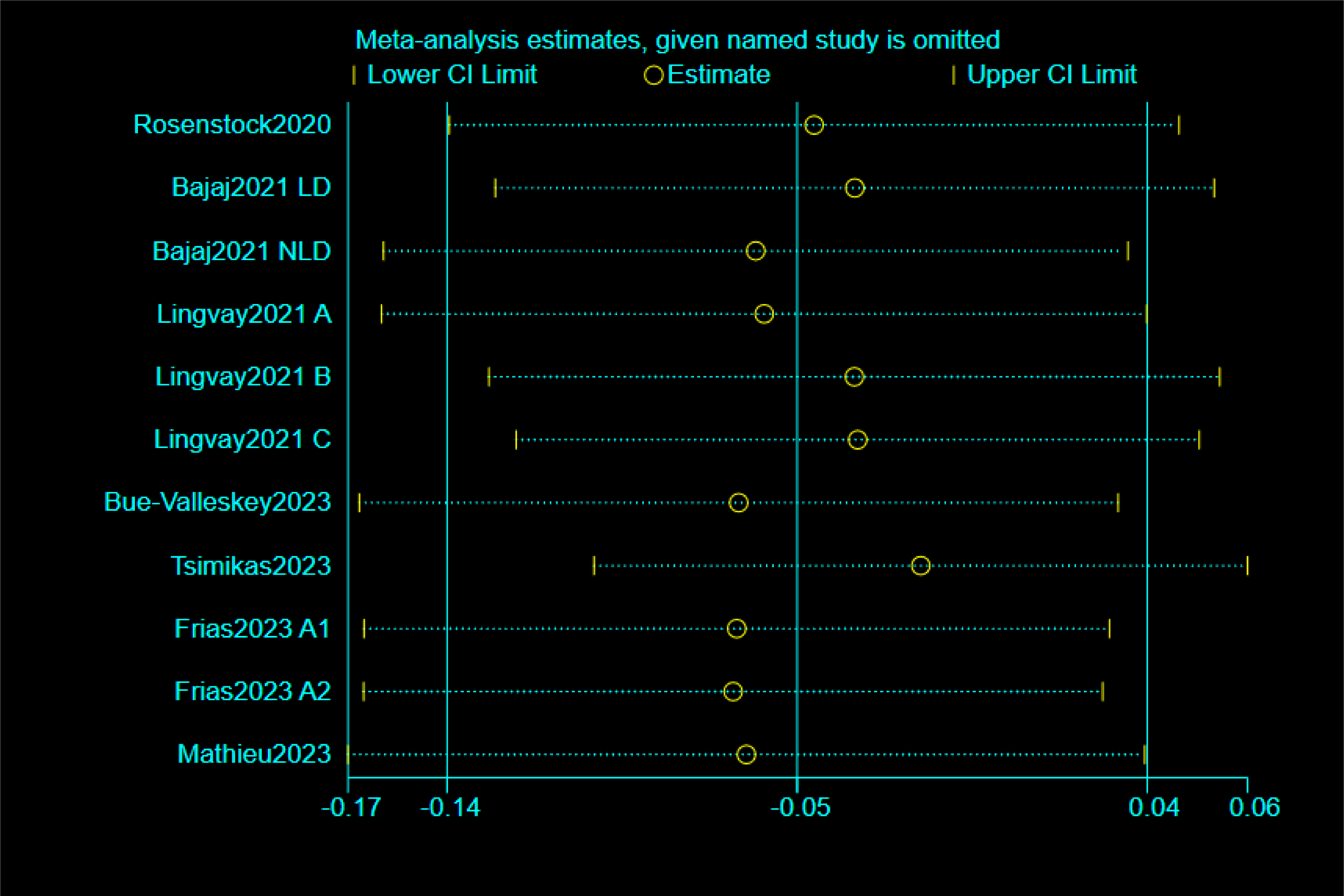

Supplement: Supplementary file 9 [file medi-102-e36308-s009.tif]

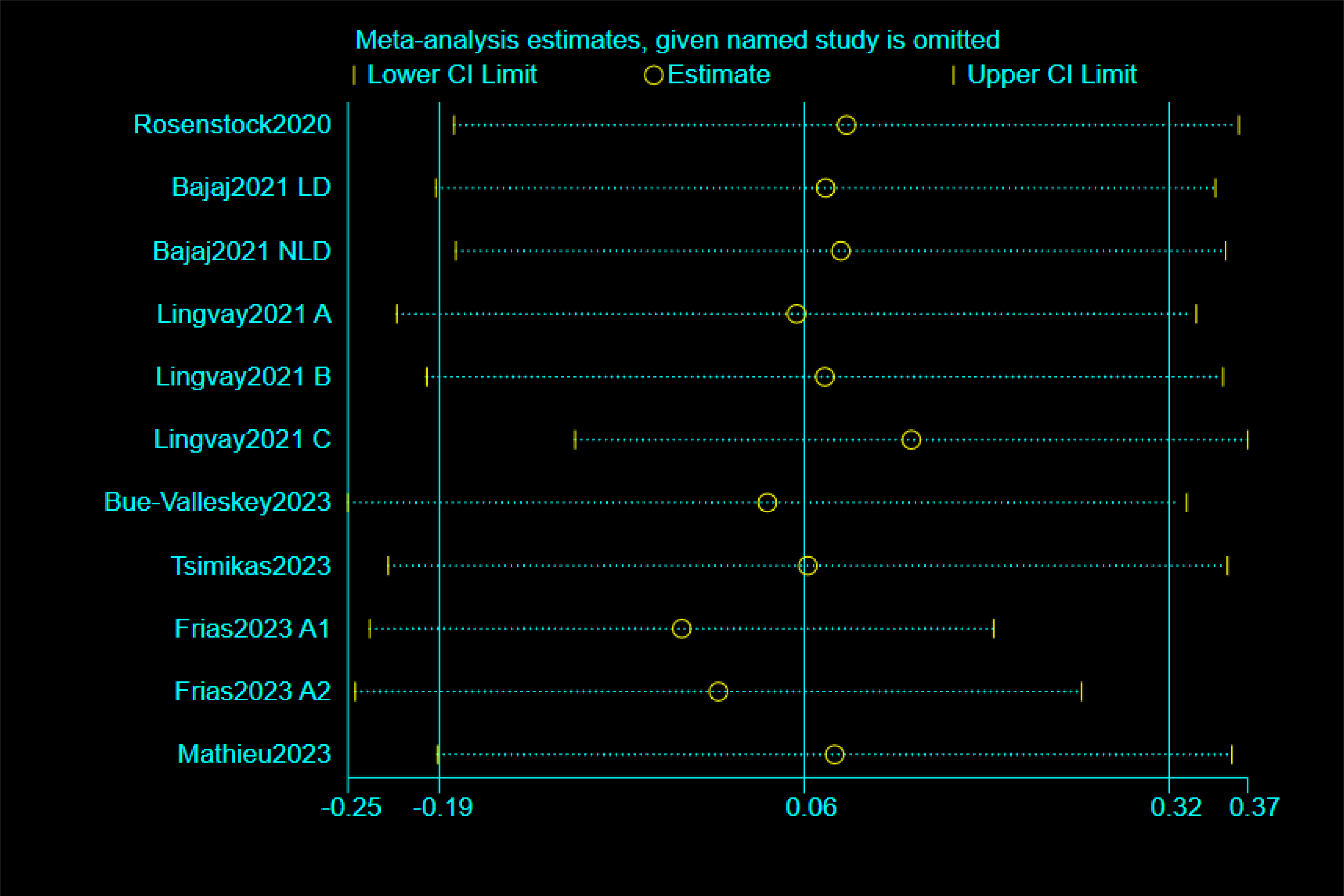

Supplement: Supplementary file 10 [file medi-102-e36308-s010.tif]

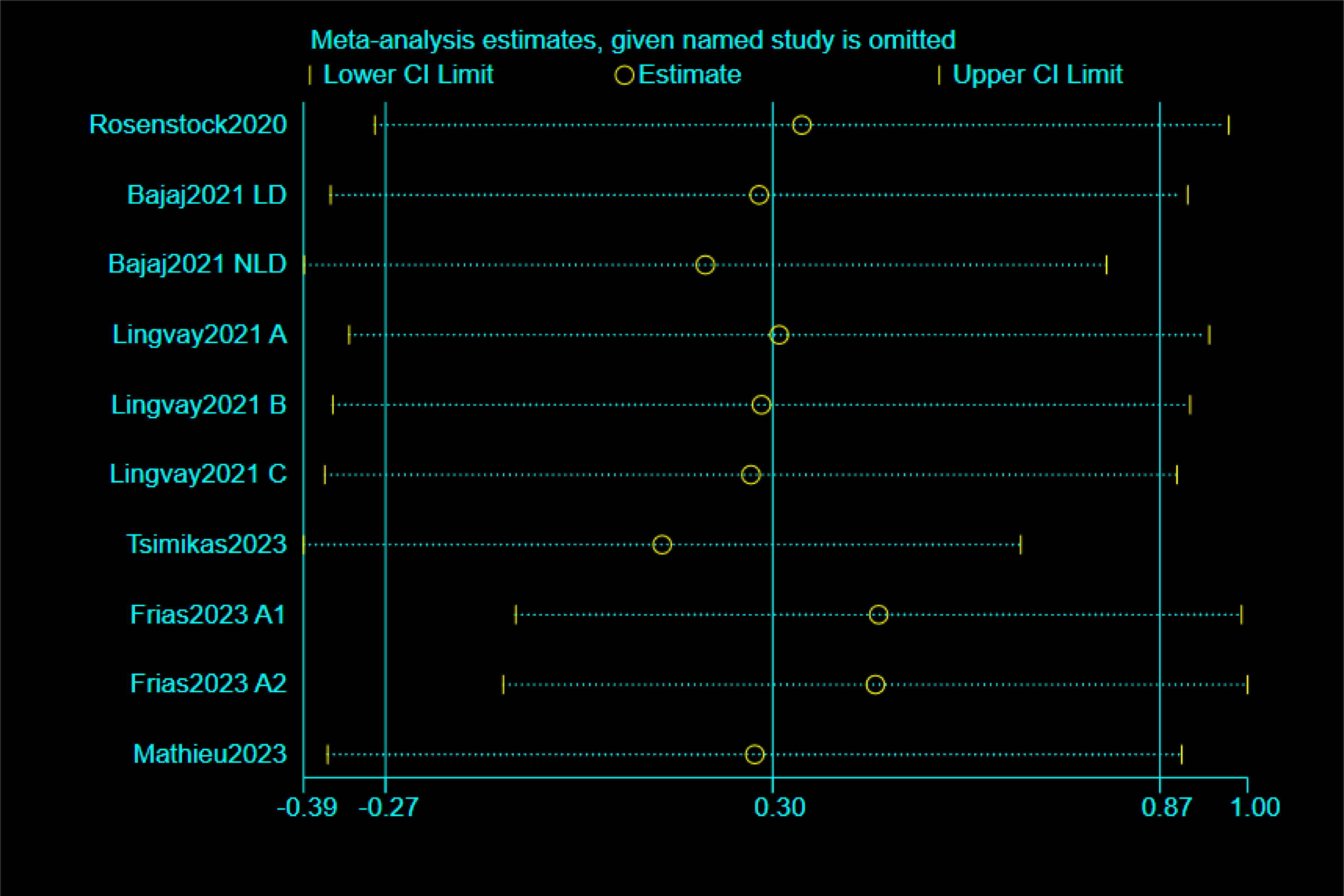

Supplement: Supplementary file 11 [file medi-102-e36308-s011.tif]

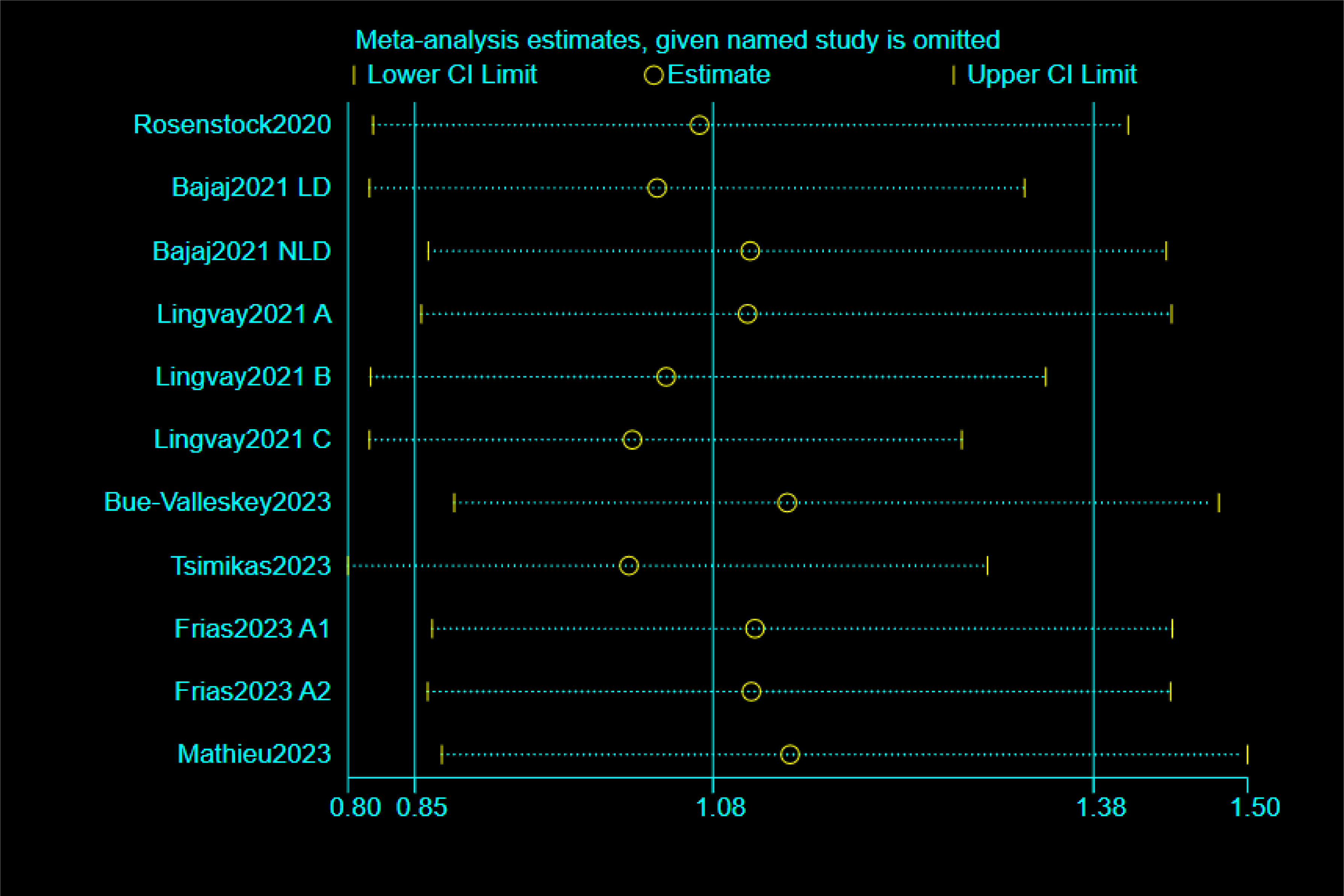

Supplement: Supplementary file 12 [file medi-102-e36308-s012.tif]

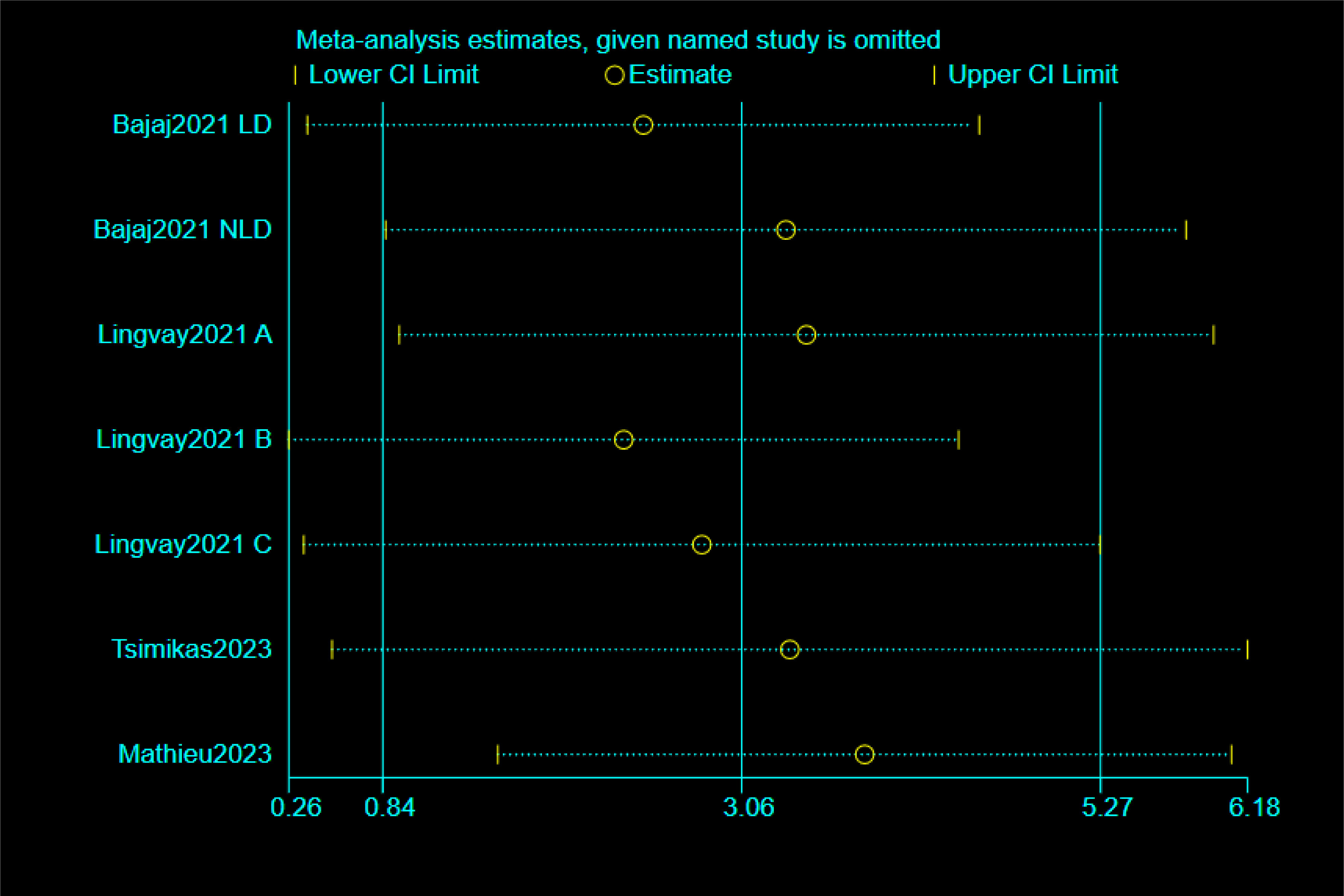

Supplement: Supplementary file 13 [file medi-102-e36308-s013.tif]

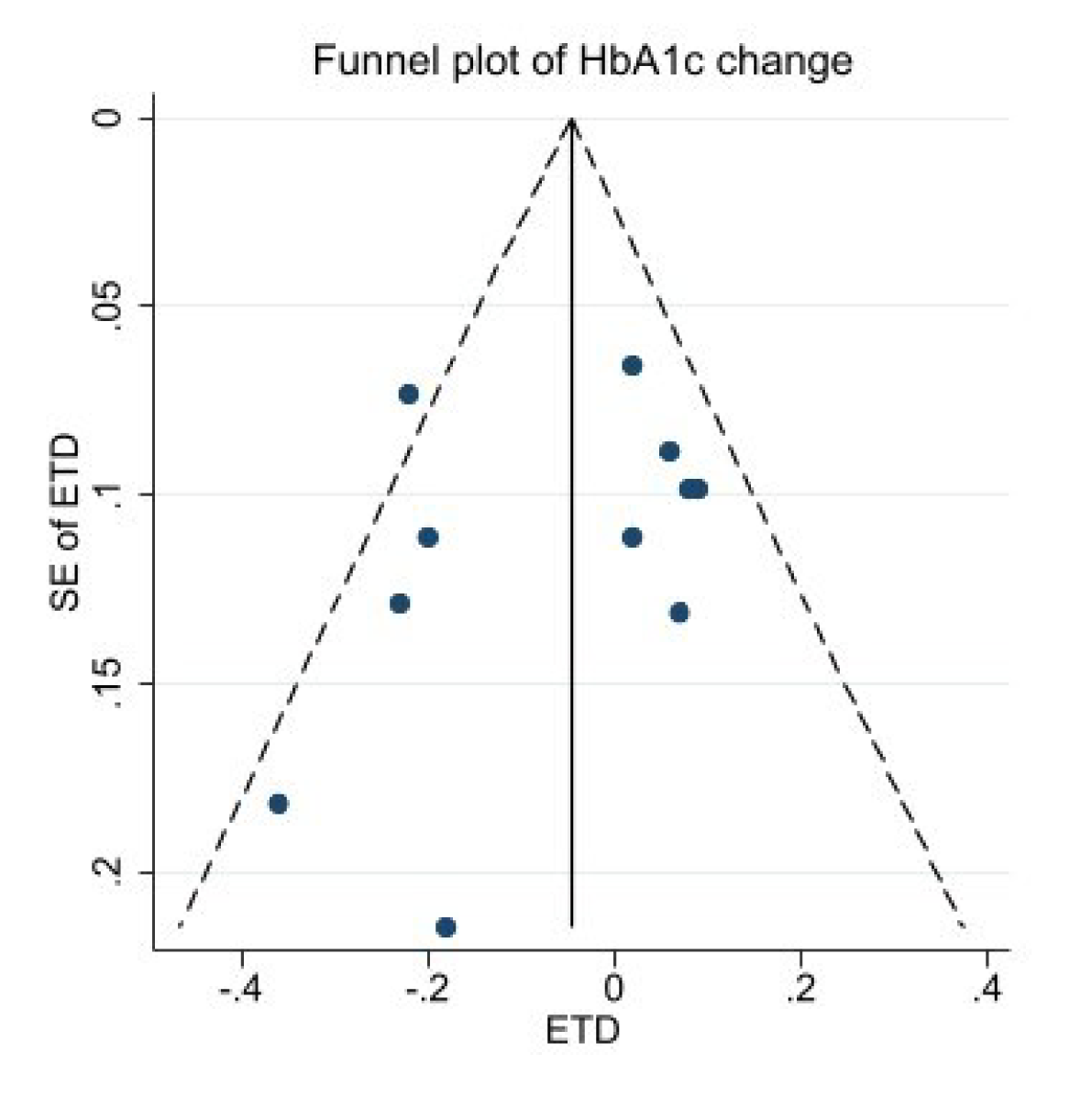

Supplement: Supplementary file 14 [file medi-102-e36308-s014.tif]

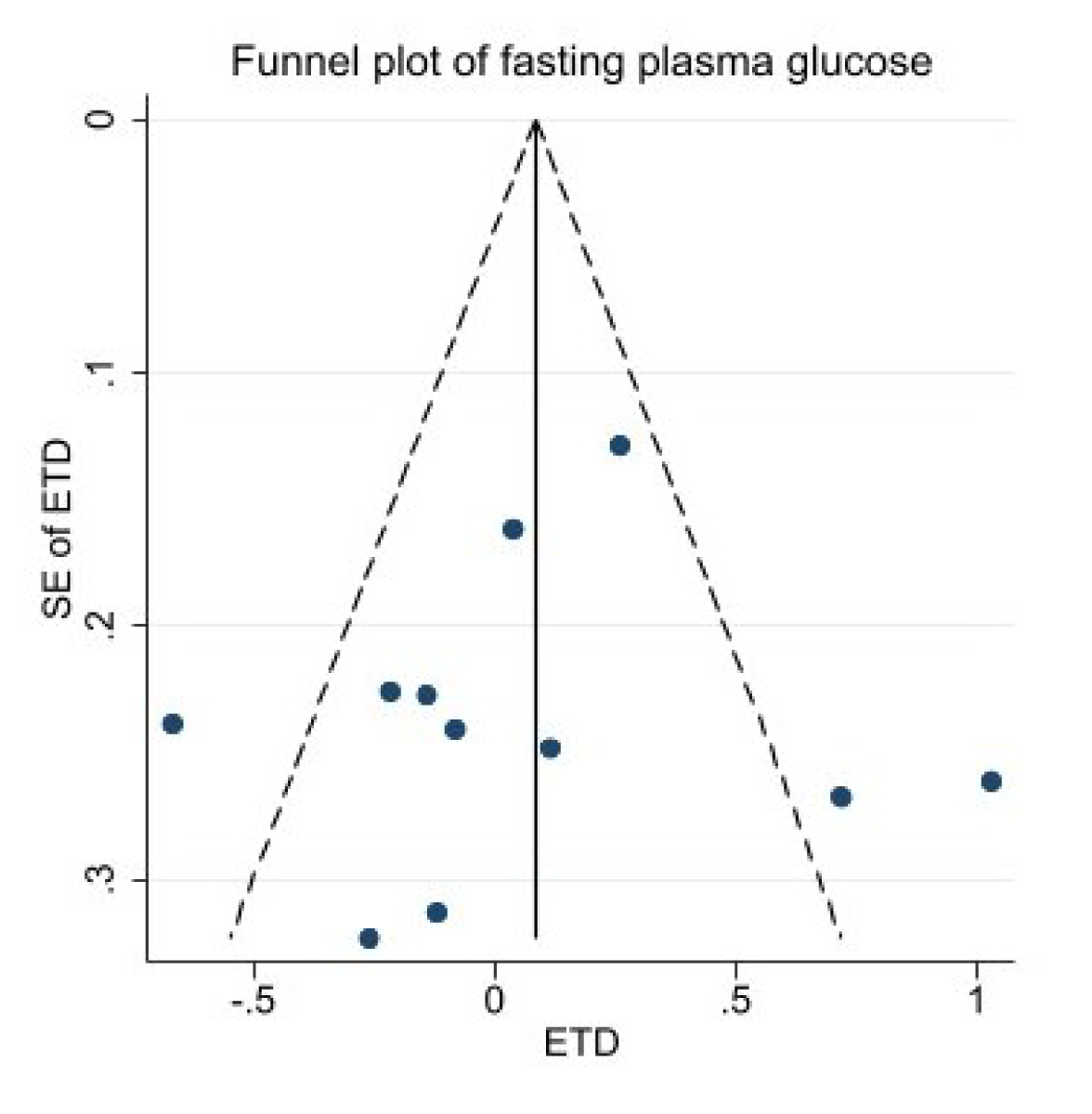

Supplement: Supplementary file 15 [file medi-102-e36308-s015.tif]

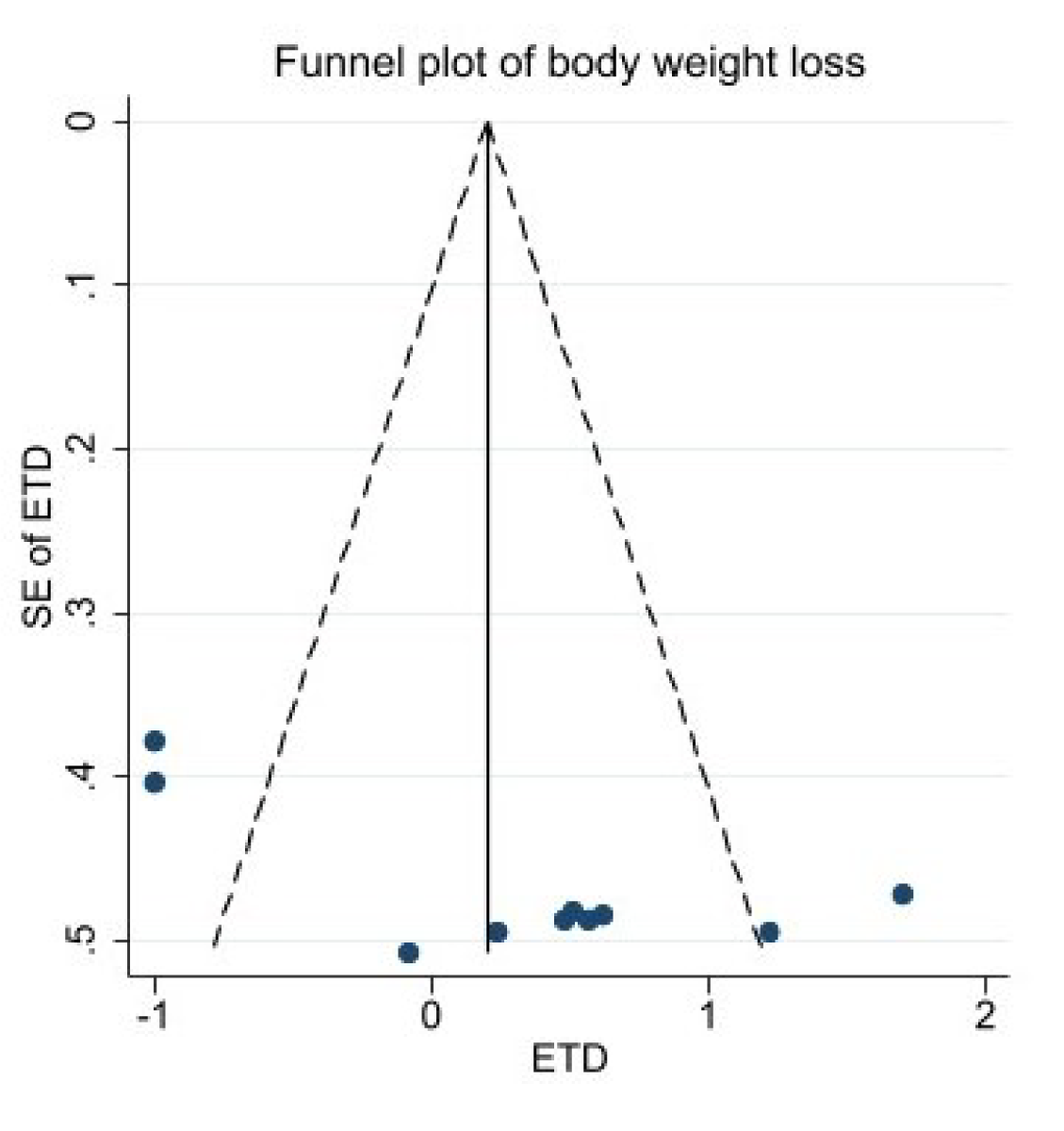

Supplement: Supplementary file 16 [file medi-102-e36308-s016.tif]

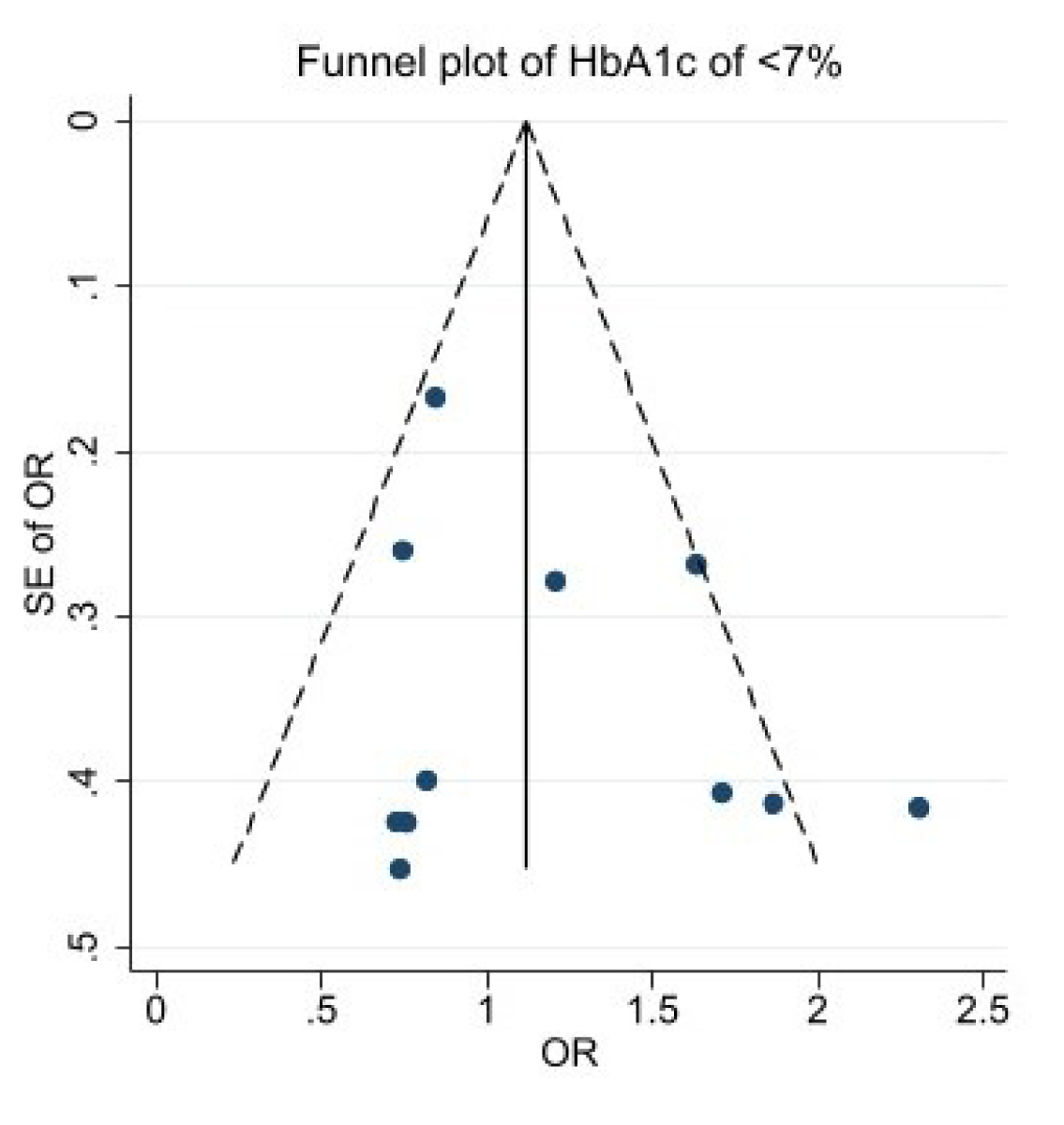

Supplement: Supplementary file 17 [file medi-102-e36308-s017.tif]

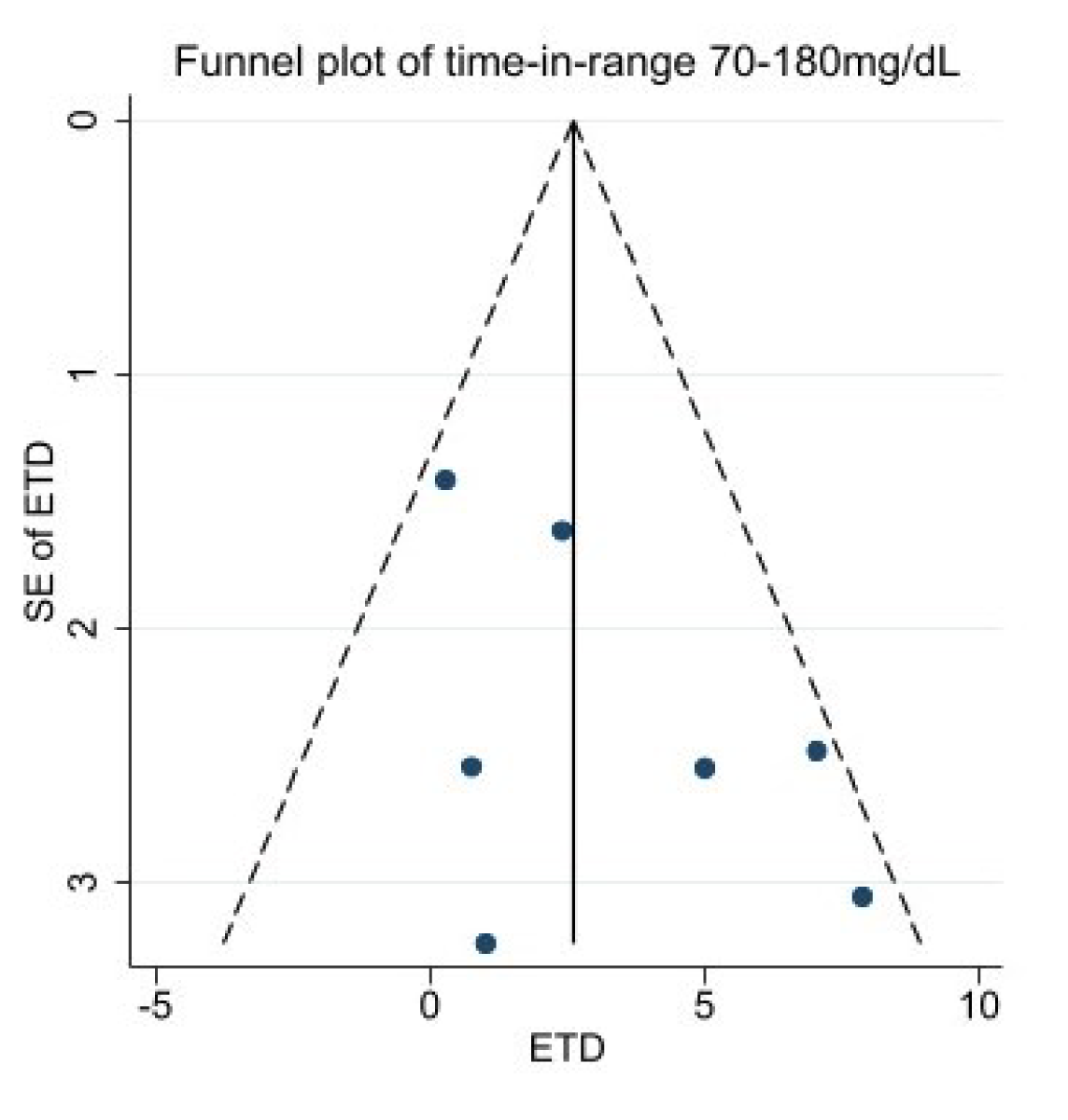

Supplement: Supplementary file 18 [file medi-102-e36308-s018.tif]
