# Supplementary material for: Efficacy and safety of once-weekly basal insulin versus once-daily basal insulin in patients with type 2 diabetes: A systematic review and meta-analysis
Source: Medicine (Baltimore). 2023 Dec 29;102(52):e36308. doi: 10.1097/MD.0000000000036308 (PMC10754560; doi:10.1097/MD.0000000000036308)
Supplement: Supplementary file 19 [file medi-102-e36308-s019.docx]

**eTable3.** Results of Egger's test

| **Outcome** | **P value** | **Publication bias** |
| --- | --- | --- |
| HbA_1c_ change | 0.449 | No |
| FPG | 0.688 | No |
| Body weight | 0.009 | Yes |
| TIR | 0.164 | No |
| HbA_1c_ < 7% | 0.289 | No |
